# Genetic variation associated with depression in Latin American populations: a systematic review of single-nucleotide variants

**DOI:** 10.3389/fpsyt.2026.1811491

**Published:** 2026-06-12

**Authors:** Gisela Aguirre, Ana Ramírez, Oscar López-Franco, Rossana C. Zepeda, Tania Molina-Jiménez, Armando Jesús Martínez, Claudia Juárez-Portilla, Mónica Flores-Muñoz

**Affiliations:** 1 Laboratorio de Neurobiología de la Conducta y Procesos Neuroquímicos, Centro de Investigaciones Biomédicas, Universidad Veracruzana, Xalapa, Veracruz, Mexico; 2 Instituto de Ciencias de la Salud, Universidad Veracruzana, Xalapa, Veracruz, Mexico; 3 Laboratorio de Biomedicina Integral y Salud, Centro de Investigaciones Biomédicas, Universidad Veracruzana, Xalapa, Veracruz, Mexico; 4 Instituto de Neuroetología, Universidad Veracruzana, Xalapa, Veracruz, Mexico

**Keywords:** ethnicity, genetic susceptibility, monoaminergic neurotransmission, personalized medicine, precision psychiatry

## Abstract

**Introduction:**

Depression is a significant global health burden, with low- and middle-income countries disproportionately affected due to stigma, limited mental health resources, and imprecise diagnosis and treatment. In Latin America, reliance on non-specialized care further contributes to heterogeneous clinical outcomes. Although personalized medicine offers opportunities to improve depression management, genetic research has largely underrepresented Latin American populations, limiting understanding of ancestry-specific risk and treatment response.

**Methods:**

This PRISMA 2020-compliant systematic review identified clinical studies assessing single-nucleotide polymorphisms in individuals of Latin American ancestry with depression through searches of PubMed, Web of Science, and EBSCO. Methodological quality was assessed, and findings were synthesized narratively due to substantial heterogeneity across studies.

**Results:**

Forty-five studies encompassing 26 cohorts (15 from Mexico, 9 from the United States, 1 from Brazil, and 1 from Peru) reported 306 variants, of which 14 were replicated across at least two independent cohorts. Variants in SLC6A4 (rs25531) and COMT (rs4680) were the most consistently reported. Low-expression rs25531 alleles were uncommon in Mexican and Mexican-American populations but more frequent among individuals with African ancestry. The COMT Met allele was repeatedly associated with greater symptom severity, increased suicide risk, and poorer response to selective serotonin reuptake inhibitors. Additional variants in TPH2, APOE, and BDNF showed ancestry- and context-dependent associations.

**Discussion:**

Despite limitations preventing meta-analysis, this review identifies both shared and population-specific genetic factors associated with depression in Latin American populations, highlighting the need for systematic and inclusive psychiatric genetics research to support the development of personalized interventions in underrepresented and resource-limited settings..

## 1 Introduction

Depression is a mental disorder regarded as a public health problem affecting up to 5% of the population, ranking as the fourth leading cause of morbidity worldwide and the primary cause of disability globally, with significant economic repercussions. The World Health Organization points to depression as the leading disabling disease in 2030 (1). Despite the well-documented symptomatology for diagnosing depression in the Diagnostic and Statistical Manual of Mental Disorders (DSM-5), diagnostic accuracy remains highly variable, and treatment choices are often empirical. With over 30 available antidepressants, their effectiveness may take up to 8 weeks to manifest, and they are effective in only about 50% of cases (2), making depression treatment unpredictable over the past five decades.

Recognized as a multifactorial and heterogeneous disorder, depression presents challenges in diagnosis and treatment (3). Genetic predispositions to the disease have been observed in 31–42% individuals with depression, compared to healthy controls (4). Clinical observations since the 1950s noted that individuals exhibit differential drug responses, suggesting a heritable component. Decades of research have confirmed that genetic factors influence both disease susceptibility and pharmacological responses, including drug efficacy and toxicity. Moreover, the frequency of genetic polymorphisms varies significantly among human populations. Importantly, allele frequencies are not randomly distributed but follow ethnic, racial, and geographical patterns, underscoring the interplay between environmental and genetic factors (5, 6).

Most patients with early or less severe depressive episodes remain untreated or are managed by non-specialized healthcare providers. Mental disorders are highly stigmatized, and more than 75% of people in low- and middle-income countries receive no treatment (7, 8). This leads to inconsistent and often inadequate treatment responses. Furthermore, most studies on depression diagnostics and predictive biomarkers have been conducted in non-Latino populations, leaving a substantial gap in understanding the genetic and pharmacogenomic diversity within Latin American cohorts.

For consistency, this review uses the terms “Latino” and “Latin American” to refer to populations with ancestry from Latin America. Although terms such as “Hispanic”, “Latino”, and “Latin American” are not strictly interchangeable, a unified terminology is adopted due to heterogeneity across studies. These terms refer to the same populations as defined in the original publications, while specific descriptors (e.g., “Mexican-American”) are retained when referring to explicitly defined cohorts.

Depression arises from interactions between genetic, biological, psychological, and environmental determinants. Traditionally, it has been associated with dysregulation of monoaminergic neurotransmission, particularly involving serotonin, norepinephrine, and dopamine. For instance, genetic variation in tryptophan hydroxylase-2 (TPH2) influences serotonin synthesis, while polymorphisms in catechol-O-methyltransferase (COMT) affect dopamine availability and cognitive control (9, 10). Beyond monoaminergic systems, additional molecular pathways are now recognized as key contributors to mood regulation and treatment response. The μ-opioid receptor (OPRM1) modulates affective processing and stress responsivity through endogenous opioid signaling, and the brain-derived neurotrophic factor (BDNF) gene, a major regulator of synaptic plasticity and neurogenesis, has been linked to antidepressant response and resilience to stress (11, 12). Similarly, apolipoprotein E (APOE) variants influence lipid metabolism, neuronal repair, and neuroinflammation, processes increasingly implicated in mood disorders (13). Accumulating data indicate that phosphodiesterase genes modulate intracellular cyclic−nucleotide levels (cAMP, cGMP) and thereby affect risk for major depression and response to antidepressants (14, 15).

Together, these findings suggest that depression results from the convergence of monoaminergic dysfunction with alterations in neuroplastic, neuroendocrine, inflammatory, and intracellular signaling pathways, reflecting a complex network of gene–environment interactions shaping individual vulnerability and treatment outcomes. Despite this growing evidence, interpretation is limited by heterogeneity and potential bias in the literature. Studies differ in single-nucleotide polymorphisms (SNPs) selection, cohort characteristics, clinical phenotyping, and outcome measures, which complicate cross-study comparisons. Moreover, variability in study design, sample size, and methodological rigor introduces a potential risk of bias, particularly in non-randomized and observational studies. By acknowledging these limitations, we aim to provide a critical synthesis of SNPs associated with depression and treatment response in individuals of Latin American ancestry, highlighting both current evidence and gaps that must be addressed to advance precision psychiatry in this population. Therefore, the objective of this systematic review was to identify and synthesize shared and population-specific SNPs associated with depression in individuals of Latin American ancestry.

## 2 Methods

### 2.1 Study design and search strategy

This systematic review was conducted following the Preferred Reporting Items for Systematic Reviews and Meta-Analyses (PRISMA) 2020 statement (16). PubMed, Web of Science, and EBSCO databases were selected to ensure broad coverage of biomedical and multidisciplinary literature. Databases were searched to identify all clinical studies investigating the association between SNPs and depression, published through January 2025. In addition, the reference lists of relevant studies were examined during the review process to ensure that key articles were not overlooked; however, no further studies meeting the predefined inclusion criteria were identified. Search terms were organized into three conceptual domains (1): participant ancestry (“Mexico”, “Latin”, “Hispanic”, “Central America”, “South America”, “Mexican”) (2); depressive phenotypes (“depression”, “depressive disorder”, “major depressive disorder”, “persistent depressive disorder”) (3); genetic variation (“mutation”, “polymorphism”, “delete”, “insert”, “mutant”, “candidate genes”, “genetic variant”, “genome-wide association study”, “GWAS”). Country-specific terms (e.g., “Mexico”, “Mexican”) were included to improve search sensitivity, as studies are frequently indexed using national rather than regional descriptors. Studies that did not meet eligibility criteria were considered only for contextual interpretation when relevant. Boolean operators were used to combine terms across domains. Full search strategies for each database, including applied limits, are provided in **Supplementary Table 1**.

### 2.2 Eligibility criteria and study selection

Records were eligible for inclusion if they were peer-reviewed articles published in English or Spanish that reported associations between SNPs and depression or depressive symptoms in human populations of Latin American ancestry. Included studies assessed depressive phenotypes in participants diagnosed with depression and involved genotyping of biological samples from affected individuals. The primary focus of the study (e.g., treatment response, comorbid conditions, or other clinical outcomes) was not a criterion for exclusion, provided that genetic associations involving SNPs and depressive phenotypes were reported.

Excluded were reviews, systematic reviews, meta-analyses, and pre-clinical studies (including *in vitro* or animal models), unpublished literature (conference abstracts, dissertations, or preprints), and studies with insufficient or incomplete data. References of included studies were hand-searched to identify additional relevant articles.

Study selection followed a multi-step screening process. Two reviewers independently screened titles and abstracts of all retrieved records to assess eligibility. Full-text articles were independently reviewed by the same two reviewers against the predefined inclusion and exclusion criteria. Disagreements at any stage were resolved through discussion, and when consensus could not be reached, a third reviewer adjudicated. No automation tools were used in the study selection process.

To meet the ancestry criteria, study participants were required to fulfill at least one of the following conditions: (i) birth in a Latin American country; (ii) birth outside Latin America with at least three grandparents born in Latin America; or (iii) self-identification as Latino.

### 2.3 Depression phenotypes and diagnostic assessment

Information regarding the definition and assessment of depression was extracted from each study, with particular attention to Major Depressive Disorder (MDD) and other depressive phenotypes. MDD was generally defined using standardized diagnostic criteria requiring the presence of acute depressive episodes lasting at least two weeks.

Persistent Depressive Disorder (PDD) was also considered when explicitly defined in the original studies according to the Diagnostic and Statistical Manual of Mental Disorders, Fifth Edition (DSM-5) (17, 18), or in earlier studies, DSM-IV definitions of dysthymic disorder, requiring a depressed mood persisting for at least two years in adults (or one year in children and adolescents), with no symptom-free period longer than two consecutive months.

Studies were further categorized based on how depression was defined in the original publications. Specifically, studies were considered to include MDD patients when a formal clinical diagnosis was established using standardized diagnostic criteria (e.g., DSM or nternational Classification of Diseases, ICD), confirmed through structured or semi-structured clinical interviews (e.g., MINI, SCID, CIDI), or when validated diagnostic algorithms were explicitly applied and reported by the original authors. Screening instruments such as the Patient Health Questionnaire-9 (PHQ-9) or PHQ-8 were considered indicative of MDD only when a diagnostic algorithm consistent with DSM criteria was explicitly applied and reported. Otherwise, studies using these instruments to assess symptom severity, score thresholds, or categorical symptom levels were classified as reporting depressive symptomatology.

For consistency in reporting, studies meeting formal diagnostic criteria were described as including MDD, whereas studies relying on screening tools or symptom-based measures without explicit diagnostic validation were described as reporting depression or depressive symptoms, in accordance with the terminology and methodological approach of each study. Other diagnostic instruments and assessment tools, including structured clinical interviews, validated diagnostic scales, or physician diagnoses, were recorded as reported. Diagnostic classification was based exclusively on information explicitly provided in the original publications; no reclassification was performed when diagnostic criteria were insufficiently described.

### 2.4 Genetic variant identification and harmonization

When reported genetic variants lacked a reference SNP cluster identifier (rsID), the dbSNP database maintained by the National Center for Biotechnology Information and the SNPedia database were consulted to retrieve and verify the corresponding identifier. Studies were excluded if they did not report specific SNPs, or variants were identified solely by sequence-based notations (e.g., NT_023935.17_16593339), which may correspond to rare, unvalidated, or non-standardized polymorphisms. Likewise, SNPs reported as deviating from Hardy–Weinberg equilibrium (HWE) in the study populations were excluded from the analysis.

### 2.5 Sample size considerations

For each included study, the sample size corresponded to the number of individuals with depression who were successfully genotyped for the variants of interest. When a study enrolled a larger number of participants but only a subset underwent genotyping, the subset that was genotyped was considered the effective sample size; for example, if 300 participants were enrolled but only 40 were genotyped, the reported sample size (n) was 40. In studies reporting multiple cohorts, ancestry-stratified analyses, or analytically independent subpopulations, sample size was recorded separately for each distinct group.

No minimum sample size threshold was applied as an inclusion criterion. Sample size was documented to facilitate descriptive comparison across studies and to support qualitative interpretation of the consistency and robustness of reported associations.

### 2.6 Data extraction

Data extraction was conducted independently by two reviewers using a structured data collection form. Extracted variables included authorship, year of publication, article title, study design, presence and type of control group, sample size, diagnostic criteria, and assessment tools for depression, depression subtype, genetic variants analyzed (SNP), and reported association with depression risk, including odds ratios (ORs) and 95% confidence intervals (CIs) when available. Disagreements were resolved by consensus or consultation with a third reviewer.

Studies were also categorized according to their primary outcome as either investigating genetic susceptibility to depression or pharmacogenetic response to antidepressant treatment.

All SNPs identified across the included studies were compiled into a structured database. To prevent double-counting of genetic signals derived from overlapping samples, each study cohort was assigned a unique cohort identifier (Cohort ID). Publications reporting data from the same underlying cohort were grouped under the same Cohort ID, while studies labeled as independent were assigned a distinct identifier, even when the same numerical cohort label was used across different articles. This approach allowed differentiation between truly independent samples and repeated analyses of shared cohorts.

SNPs were standardized using rsID identifiers and annotated with study-level characteristics, including author, publication year, Cohort ID, sample size, control type, availability of odds ratios, absolute odds ratio values, confidence interval bounds, ancestry classification, depression subtype, study design, and subpopulation. SNPs reported across multiple cohorts were retained and flagged accordingly to allow assessment of recurrence across independent and overlapping samples.

Due to substantial heterogeneity in study design, SNP reporting, and cohort overlap, meta-analysis was not performed.

All data processing and cross-study comparisons were conducted using R version 4.5.2 for Windows (64-bit) (19) and RStudio Desktop (20). Genome-wide association summary statistics were processed using the data.

Nominal significance was defined as P < 0.05, based on the reported P-values corresponding to the summary statistics.

### 2.7 Risk of bias assessment

Methodological quality was independently assessed by two reviewers, with disagreements resolved through discussion and consensus. Assessment tools were selected based on the study design.

Randomized Controlled Trials (RCTs) were evaluated using the Revised Cochrane Risk of Bias (RoB) tool for randomized trials (RoB2) (21), which assesses bias across five domains: randomization process, deviations from intended interventions, missing outcome data, outcome measurement, and selective reporting. The overall rating was determined by the highest level of concern identified in any domain.

Non-randomized controlled studies (non-RTCs) were assessed using the Risk Of Bias In Non-randomized Studies of Interventions, Version 2 (ROBINS-I V2) (22), which evaluates bias across seven domains, including confounding, classification of interventions, selection of participants, deviations from intended interventions, missing data, measurement of outcomes, and selective reporting. Overall risk of bias reflected the most severe domain rating, and studies classified as “critical risk” were excluded from the synthesis.

Observational studies, including case–control and cohort designs, were assessed using the Newcastle–Ottawa Scale (NOS) (23). Studies were scored on a nine-point scale and categorized as high quality (7–9 points), moderate quality (4–6 points), or low quality (0–3 points).

One study reported results from both randomized and non-randomized analyses. Each component was assessed independently using the tool appropriate to its design to preserve methodological rigor.

For visualization, risk-of-bias categories were harmonized across tools into three levels (1): low risk/high quality (2); moderate risk/some concerns; and (3) high risk, serious or critical risk/low quality. The proportion of studies in each category was calculated and presented graphically. No automation tools were used in risk-of-bias assessment; all judgments were based solely on information reported in the original publications.

## 3 Results

### 3.1 Study selection

**Figure 1:**
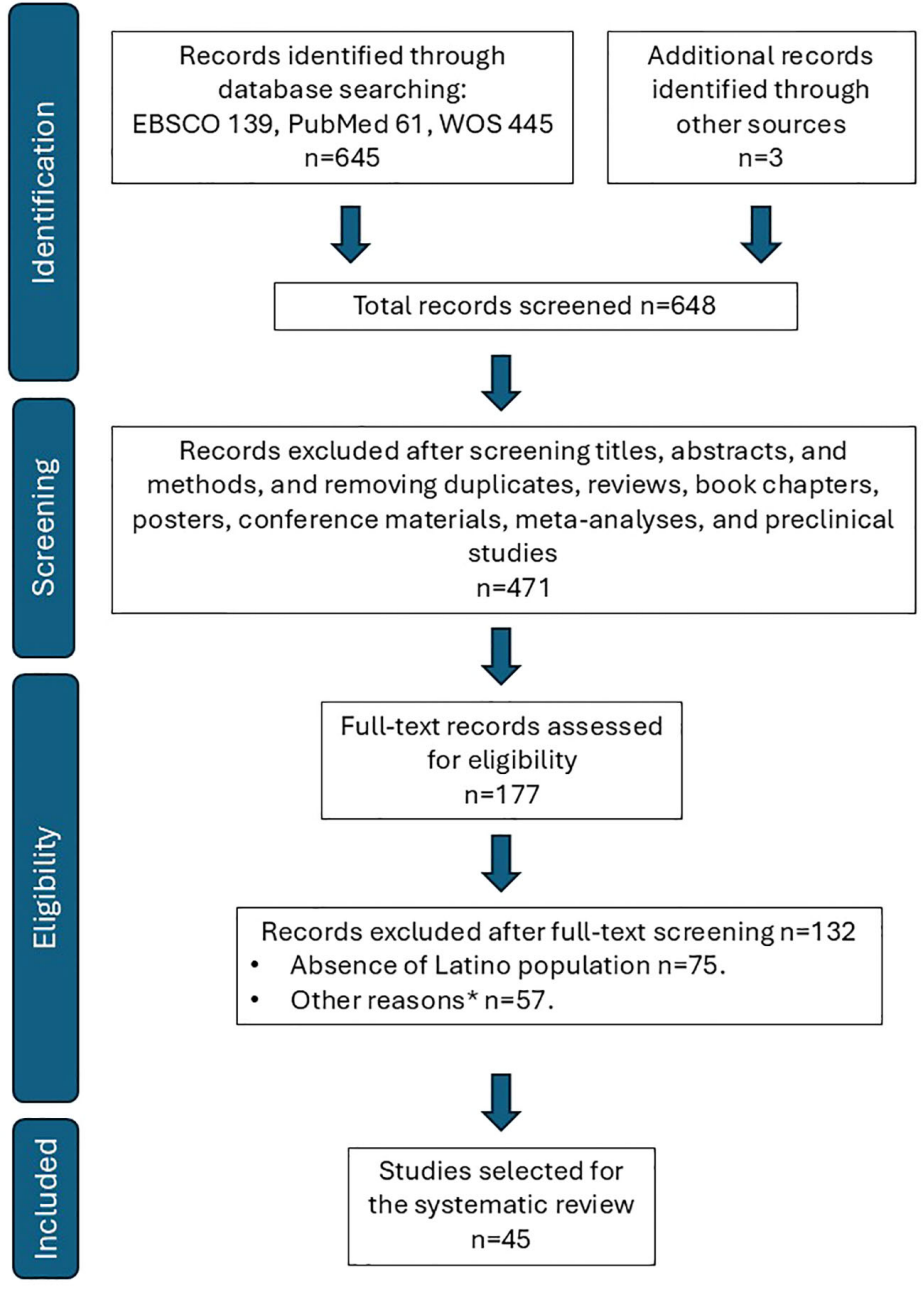
PRISMA-flow diagram. Flowchart of the records included passing he screening and selection process for the systematic review on SNPs and depression in Latino populations. *Absence of depressed individuals, lack of SNP reporting.

Using the described methodology, 645 records were identified across different sources: 139 from EBSCO, 61 from PubMed, and 445 from Web of Science. Additionally, 3 records were manually added, bringing the total to 648. After excluding duplicates, reviews, books, posters, book chapters, conference materials, meta-analyses, and pre-clinical models, 177 records remained. A detailed evaluation of abstracts and, when necessary, full-text methodologies was conducted, and records were further excluded based on non-Latino populations (75 records) and other reasons (57 records), including absence of depressed participants, lack of SNP data, or failure to report genetic associations, resulting in 132 records being eliminated after the second round of screening. Ultimately, 45 records were included in the systematic review (**Figure 1**). All included studies involved individuals of Latino ancestry with diagnosed or explicitly assessed depressive phenotypes and investigated single-nucleotide polymorphisms (SNPs). No additional search strategies were employed to supplement the study selection.

### 3.2 Study characteristics

The included studies addressed two distinct types of outcomes: (i) genetic susceptibility to depression, including risk, symptom severity, and related clinical phenotypes, and (ii) pharmacogenetic predictors of antidepressant treatment response. These outcome domains were considered separately in the synthesis to avoid conflating distinct biological and clinical questions.

#### 3.2.1 Study design and control groups

Of the 45 studies included in this review, 15 evaluated clinical interventions: 8 randomized controlled trials (RCTs), 6 non-randomized controlled studies (non-RCTs), and 1 study reporting both a randomized and a non-randomized analysis. The remaining 30 studies were observational in design, comprising 12 case-control studies and 18 cohort or descriptive studies (**Table 1**).

**Table 1:** Summary of study design and parameters considered in the articles.

INTERVENTIONAL STUDIES			
Reference	Remarks	Design	Risk of Bias
(24)	Fluoxetine and Desipramine treatment. SNP Association with Remission of Depression.+**	RCT	High
(25)	Fluoxetine and Desipramine treatment. SNP Association with Diagnosis/Remission of Depression.+**	RCT	Moderate
(26)	Fluoxetine and Desipramine treatment. SNP Association with Diagnosis/Remission of Depression.+**	RCT	Low
(27)	Fluoxetine and Desipramine treatment. SNP Association with Remission of Depression.+**	RCT	Low
(28)	Fluoxetine and Desipramine treatment. SNP Association with Remission of Depression.+**	RCT	High
(29)	Fluoxetine and Desipramine treatment. SNP Association with Remission of Depression.+**	RCT	Moderate
(30)	Fluoxetine and Desipramine treatment. SNP Association with Diagnosis/Remission of Depression.+**	RCT	Low
(31)	Fluoxetine and Desipramine treatment. SNP Association with Diagnosis/Remission of Depression.+**	RCT	High
(32)	Fluoxetine, desipramine or citalopram treatment. SNP Association with Remission of Depression.+** Compares results from Wong et al., 2006 (RCT) and phase 1 of STAR*D (N-RCT) sub-samples.	RCT + N-RCT	Moderate
(33)	Citalopram treatment. SNP Association with Diagnosis/Remission of Depression.+**	N-RCT	High
(34)	Citalopram treatment. SNP Association with Remission of Depression.+**	N-RCT	High
(35)	Fluoxetine treatment. SNP Association with Remission of Depression.+***	N-RCT	High
(36)	Fluoxetine treatment. SNP Association with Remission of Depression.+***	N-RCT	High
(37)	Citalopram treatment. SNP Association with Remission of Depression.+**	N-RCT	High
(38)	HCV patients with comorbid Depression by IFNα treatment.+***	N-RCT	High
OBSERVATIONAL STUDIES: CASE CONTROL			
Reference	Remarks		Risk of Bias
(39)	Obsessive-Compulsive Disorder (OCD) with comorbid Depression associated to MAO-A SNPs.+*		Low
(40)	BDNF SNP and depression.+**		Low
(41)	Childhood psychosocial adversities on Depression associated with BDNF SNP and SLC6A4 SNPs.+*		High
(42)	SNP Association with Diagnosis.*		High
(43)	Pregnancy Depression associated with TNF-α and SNPs.*		High
(44)	Suicide risk associated with CYP46A1 SNPs.+*		High
(45)	Adverse Childhood Experiences on the Severity of Depression associated with MAO SNPs.+*		High
(46)	SNP Association with Diagnosis.+**		High
(47)	SNP Association with Diagnosis.+**		High
(48)	APOE SNPs and association with Alzheimer’s Disease and with Anxiety and Depression.**		High
(49)	SNP Association with Diagnosis.+**		High
(50)	HIV patients with comorbid Depression associated with TPH2 SNPs.*		High
OBSERVATIONAL STUDIES: COHORTS			
Reference	Remarks		Risk of Bias
(51)	DISC1 variation on neuroanatomical and neurocognitive phenotypes.+**		High
(52)	Chronic kidney disease patients with comorbid Depression associated with IL-6 and IL-10 SNPs.*		High
(53)	SNP Association with Diagnosis of Depression.**		High
(54)	SNP Association with Diagnosis of Depression.**		Moderate
(55)	TPH1 SNP associated with suicide attempt.+**		High
(56)	Susceptibility to developing stress and depression.**		High
(57)	SNP association with cannabbis use and depression.+**		High
(58)	CMPT SNP association with depression.**		High
(59)	SNP Association with Diagnosis of Depression.+**		High
(60)	Estrogen receptor SNP in post-menopausal women.**		High
(61)	SNP Association with Diagnosis of Depression.+**		High
(62)	ANKK1, DDR4, and GRIN2B SNPs association with children and adolescents behavior.**		High
(63)	PTPN22 associated with frailty.**		High
(64)	Nondemented carriers of presenilin-1 mutations: autosomal dominant pattern of inheritance of early-onset dementia consistent with AD.**		High
(65)	Depression associated to presenilin-1 SNP in women.**		High
(66)	Childhood maltreatment association with Depression and 5-HTTLPR genotype.**		Moderate
(67)	SNP association with suicide attempt in depressed adolescents.+**		High
(68)	SNPS associated with depression, PTSD, and suicidal ideation/self-harm.**		Moderate

Depression type: +, Major Depressive Disorder (MDD; episodes ≥2 weeks, may resolve or recur): Includes participants with a formal clinical diagnosis based on DSM or ICD criteria, confirmed through structured clinical interviews (e.g., MINI, SCID, CIDI). Unmarked = Depressive Symptomatology: Encompasses participants identified through screening scales or symptom indices (e.g., PHQ-8, PHQ-9, CES-D, BDI, or author-developed indices). This category includes terms referred to in the original studies as "depression," "depressive symptoms," or "major depression" when such labels were based solely on screening tools rather than clinical diagnostic validation. When controls were included: *psychiatric evaluation, **no psychiatric evaluation, ***no control group. RCT, Randomized Controlled Trial; N-RCT, Non-Randomized Controlled Trial; STAR*D, Sequenced Treatment Alternatives to Relieve Depression. Risk of bias was assessed by either NOS, RoB2 or ROBINS-I according to the study design.

Most clinically controlled studies included in this review evaluated treatment strategies for depression, primarily through a pharmacogenetic approach, specifically examining remission in relation to specific SNPs. Of these, four studies drew upon data from a shared cohort of participants who were treated with citalopram as part of the Sequenced Treatment Alternatives to Relieve Depression (STAR*D) trial funded by the National Institute of Mental Health of USA (32–34, 37). The two records evaluating fluoxetine were conducted using the same cohort of participants (35, 36). The eight studies assessing fluoxetine or desipramine used a common study population (25–31). Each set of studies was therefore derived from a unique, non-overlapping participant cohort (see Participant Cohorts and Sample Overlap section). Interestingly, Cabanero et al. (32) compared two samples, one derived from STAR*D participants treated with citalopram (without explicitly reporting Latino ethnicity) and another from Wong et al. (25) treated by either fluoxetine or desipramine.

One study examined depressive symptoms in patients with Hepatitis C treated with interferon-alpha (TNF-α) (38). Despite differences in primary outcomes, such as treatment remission or depression secondary to medical conditions, all studies were included because they reported SNP data derived from participants diagnosed with depression.

Among the 12 case-control studies included, 8 explicitly reported that control participants underwent psychiatric evaluation to confirm the absence of depression (39–45, 50), whereas the remaining 4 studies included control groups for whom the absence of depression was assumed but not formally assessed (46–49).

The case-control studies encompassed a wide range of comorbid conditions and examined genetic associations between depression and various clinical or psychosocial factors. One study examined the association between tumor necrosis factor-alpha (TNF-α) SNPs and depression during pregnancy, identifying inflammation-related genetic variants potentially involved in perinatal mood disorders (43). Another study assessed the risk of suicide in relation to CYP46A1 polymorphisms, examining a role for cholesterol metabolism pathways in suicidal behavior within MDD (44). The impact of adverse childhood experiences (ACEs) on the severity of MDD was investigated in relation to monoamine oxidase (MAO) gene variants, allowing examination of gene–environment interplay in depression vulnerability (45). Similarly, one study explored the role of MAO-A SNPs in individuals diagnosed with obsessive-compulsive disorder (OCD) and comorbid MDD, evaluating shared neurobiological mechanisms across these conditions (39).

The combined influence of childhood psychosocial adversities and genetic susceptibility to MDD was also examined through variants in the BDNF and SLC6A4 genes (41). In addition, three studies, based on the same cohort of participants, investigated genome-wide functional variants associated with MDD, providing broader insights into polygenic contributions to the disorder (46, 47, 49).

Several studies explored MDD in the context of comorbid medical conditions. One assessed the association between APOE gene variants and both Alzheimer’s disease and MDD and anxiety symptoms, while another evaluated MDD in relation to APOE polymorphisms independent of dementia (38, 44). Finally, one study investigated TPH2 gene polymorphisms in individuals living with HIV and comorbid depression, addressing the serotonergic mechanisms involved in mood regulation within immunocompromised populations (50).

Among the 18 cohort studies, 11 aimed to characterize genetic variants associated with depression across diverse populations (53, 54, 56–59, 61, 65–68). In contrast, 7 studies examined genetic polymorphisms in the context of other primary conditions or traits, rather than establishing direct associations with depression. These included investigations in nondemented carriers of presenilin-1 mutations consistent with an autosomal dominant pattern of early-onset Alzheimer’s disease (64), estrogen receptor polymorphisms in postmenopausal women (60), and the relationship between specific gene variants and behavioral outcomes in children and adolescents (62). Additional studies that used standardized diagnostic tools for depression explored gene polymorphisms in relation to frailty (63), neuroanatomical and neurocognitive phenotypes (51), and suicidal behavior (55). A further study explored interleukin-6 (IL-6) and interleukin-10 (IL-10) gene variants in patients with chronic kidney disease (CKD) and comorbid depression, examining the role of inflammatory pathways (52), which are well-documented to contribute to the pathophysiology of depression, particularly in individuals with chronic illness. Although these studies included participants with depressive symptoms or related outcomes, their primary objective was not to examine direct associations between SNPs and depression. Nonetheless, these studies reported SNP data derived from Latino participants with depressive symptoms or diagnoses and were therefore retained.

#### 3.2.2 Depression phenotypes and diagnostic assessment

Across the selected studies, MDD was diagnosed according to DSM-IV criteria (24–34, 37, 39–41, 45–47, 49, 51, 55) or other diagnostic frameworks reported by the original authors. Diagnostic assessments for MDD were conducted using structured or semi-structured interviews, including the International Statistical Classification of Diseases and Related Health Problems, 10th Revision (ICD-10) (51), the Mini-International Neuropsychiatric Interview (MINI) (35, 36, 44, 59, 61) and MINI-Plus (51, 57), the Composite International Diagnostic Interview (CIDI) (38), and the Kiddie Schedule for Affective Disorders and Schizophrenia for School-Age Children-Present and Lifetime Version (K-SADS-S-PL) (67).

In addition to diagnostic assessments, some studies assessed depressive symptom severity using self-report screening instruments, including the PHQ-8 (58) and PHQ-9 (52, 68), the six-item version of the Center for Epidemiologic Studies Depression Scale (CES-D) (42, 53, 54), and the Beck Depression Inventory (BDI) (50, 56, 64, 65). Clinician-rated instruments were also reported, including the Hamilton Depression Rating Scale (HAM-D) (60), the Geriatric Depression Scale (GDS) (48), and the Edinburgh Postnatal Depression Scale (EPDS) (43). Only one study developed a screening index for depressive symptoms (66), and one study used a subsample of participants where neuropsychiatric disorders were self-reported (48).

Other instruments used included the Behavior Assessment System for Children, Second Edition (BASC-II) and the Conners Parent Rating Scales (CPRS) (62). These instruments were used in studies involving children and adolescents and include subscales assessing depressive symptoms, among other behavioral and emotional domains.

Based on the diagnostic approaches described in each publication, 29 of the 45 included studies used standardized diagnostic criteria or validated diagnostic instruments to identify MDD, including structured clinical interviews or diagnostic algorithms based on DSM or ICD frameworks. In contrast, 16 studies assessed depressive symptoms using screening instruments or symptom-based measures without formal diagnostic validation (**Table 1**).

Although these studies used a variety of terms, including, “depression,” “major depression,” “depressive mood,” or related constructs; to describe the phenotype, these labels were not consistently supported by standardized diagnostic criteria and were therefore classified as depressive symptomatology for the purposes of this review.

None of the included studies explicitly defined PDD according to standardized diagnostic criteria.

#### 3.2.3 Ancestry, participant cohorts, and sample overlap

Of the 45 studies included, 19 involved participants born in a Latin American country, 13 included individuals born outside Latin America with at least three grandparents of Latin American origin, and 13 relied on self-identification as Latino. In several of these latter studies, geographic ancestry was not further specified, or participants represented admixed populations residing outside Latin America. A summary of ancestry classification and cohort characteristics is provided in **Table 2**.

**Table 2:** Participant cohorts and sample overlap across studies.

Region	Cohort id	Recruitment places	n	reference
USA	LA	Los Angeles, CA	272	(27)+
			233	(24)+
			272	(28)+
			278	(29)+
			278	(40)+
			284	(25)+*
			284	(26)+
			278	(46)+
			101	(31)+
			203	(47)+
			181	(30)+
			203	(49)+
	STAR*D	Dallas, Texas; San Diego and Los Angeles California form the STAR*D cohort	268	(32)+++*
			163	(34)++
			238	(37)++
			196	(33)++
	San Antonio	San Antonio Family Study cohort	1221	(59)++
			626	(51)++
			893	(61)++
			434	(57)+
	WHI	40 clinical centers in the United States from the Women’s Health Initiative (WHI)	3138	(53)++
	Multi-state (CA/IL/NY/FL)	San Diego, Chicago, Bronx area and Florida	12310	(54)++
	TX	Rio Grande Valley, Brownsville and McAllen, Texas	535	(48)++
	TX & CA	Dallas, Texas; Long Beach and Los Angeles California	85	(38)++
	KY	Central Kentucky	124	(58)++
	NY & PEN	NY & Pennsylvania, USA	32	(55)++
Latin America	M1	Mexico City National Institute of Psychiatry	105	(39)
		Mexico City Instituto Nacional de Neurologia y Neurocirugia	33	(64)
	M2		30	(65)
	M3	Mexico City. From the Integrated Study of Depression Among Elderly, Mexican Institute of Social Security (IMSS)	226	(42)
	M4	Mexico City. Adolescent inhabitants of the metropolitan area	246	(41)
	M5	Mexico City. Hospital Psiquiatrico Infantil Juan N. Navarro	197	(67)
	M6	Mexico City. Centro Médico Nacional 20 de Noviembre, Institute for Social Security and Services for State Workers (ISSSTE)	175	(45)
	M7	Mexico City. From the Early Life Exposures in Mexico to Environmental Toxicants (ELEMENT) cohort	590	(62)
	M8	Mexico City. National Medical Center “La Raza”, IMSS	81	(50)
	MIC	Michoacan. Patients from the Mental Health Center of Michoacán (Centro Michoacano de Salud Mental).	69	(35)
			56	(36)
	DUR	Durango. Pregnant women with depression who attended a public hospital in Durango City.	153	(43)
	GTO	Guanajuato. Post-menopausal women.	177	(60)
	VER	Veracruz. Population of the Health Center of Sanitary Jurisdiction Number 7 in Zapoapan, Ixtaczoquitlan.	98	(56)
	JAL	Jalisco. Patients diagnosed with stage 5 chronic kidney disease at the at the Hospital Regional No. 46 of the IMSS in Guadalajara City.	13	(52)
	NL	Monterrey, NL. Suicide victims and MDD patients from the “Dr. Jose Eleuterio Gonzalez” University Hospital, Autonomous University of Nuevo Leon, Monterrey.	126	(44)
	Multi-State Mx	Diverse Mexican states from the Study of Nutritional and Psychosocial Markers of Frailty: Michoacan, Mexico, Guanajuato, Veracruz, Oaxaca, Hidalgo, Puebla, Guerrero, Jalisco, Tlaxcala, Queretaro, San Luis Potosi, Yucatan, Coahuila, Tamaulipas, Zacatecas, Sinaloa, Durango, Nuevo León, Colima, Baja California, Aguascalientes, Morelos, Chihuahua and Tabasco.	630	(63)
	Brazil	Children born in Pelotas, Brazil from the Pelotas Birth Cohort Study.	80	(66)
	Peru	Participants of the Pregnancy Outcomes, Maternal and Infant Study (PrOMIS) who attended prenatal care clinics at the Instituto Nacional Materno Perinatal (INMP) in Lima, Peru.	3308	(68)

Unmarked, Participants born in a Latin American country. +, Participants born elsewhere but with at least 3 grandparents of Latin American origin. ++, Participants self-identified as Latino. +++, Participants described + and ++. MDD, Major Depressive Disorder. LA, Los Angeles. STAR*D, Sequenced Treatment Alternatives to Relieve Depression. *Cabanero et al. (32) compared samples from the STAR*D and from the LA cohorts (25). WHI, Participants in the Women's Health Initiative observational study and clinical trials. Mexico City cohorts are labeled M1–M8. DUR, Durango. GTO, Guanajuato. JAL, Jalisco. MIC, Michoacan. Multi-State Mx, Diverse Mexican states from the Study of Nutritional and Psychosocial Markers of Frailty. NL, Monterrey. VER, Veracruz. Mexico City cohorts are labeled M1–M8.

In total, 26 studies investigated Latino populations recruited in the United States. Four studies analyzed data from the Sequenced Treatment Alternatives to Relieve Depression (STAR*D) study, which included self-identified Latino participants from Texas and California (32–34, 37). Four additional studies analyzed data from the San Antonio Family Study cohort, analyzed in four articles (51, 57, 59, 61).

Twelve studies reported analyses based on a cohort of Mexican-American individuals recruited in Los Angeles, California (24–31, 40, 46, 47, 49). Subpopulation identifiers were applied during data extraction to distinguish participant origins and to avoid duplication in downstream analyses. One study (32) included two independent samples: one derived from the STAR*D cohort and another from the Mexican-American cohort previously reported by Wong et al. (25).

Dunn et al. (53) analyzed data from the SHARe cohort of the Women’s Health Initiative, which included participants recruited across 40 clinical centers in the United States. A subsequent study from the same group reported on a large Latino sample recruited from four sites, including California, Illinois, New York, and Florida (54). Additional U.S.-based Latino cohorts included samples from New York and Pennsylvania (55), Texas and California (38), Kentucky (58), and Texas (48).

Nineteen cohorts included individuals born in Latin America, comprising 17 cohorts from Mexico and two from other Latin American countries: Peru (68) and Brazil (66). The Peruvian study reported a genome-wide dataset including 1,048,575 unique SNPs, which was analyzed as an independent cohort and compared descriptively with SNPs reported in other cohorts.

Within Mexico, 15 distinct cohorts were identified. Eight unique cohorts were recruited in Mexico City (39, 41, 42, 45, 50, 62, 64, 65, 67). One study included participants from 25 Mexican states (63) Additional cohorts were recruited in Michoacan (35, 36), Guanajuato (60), Durango (43), Jalisco (52), Veracruz (56) and Nuevo Leon (44).

**Figure 2:**
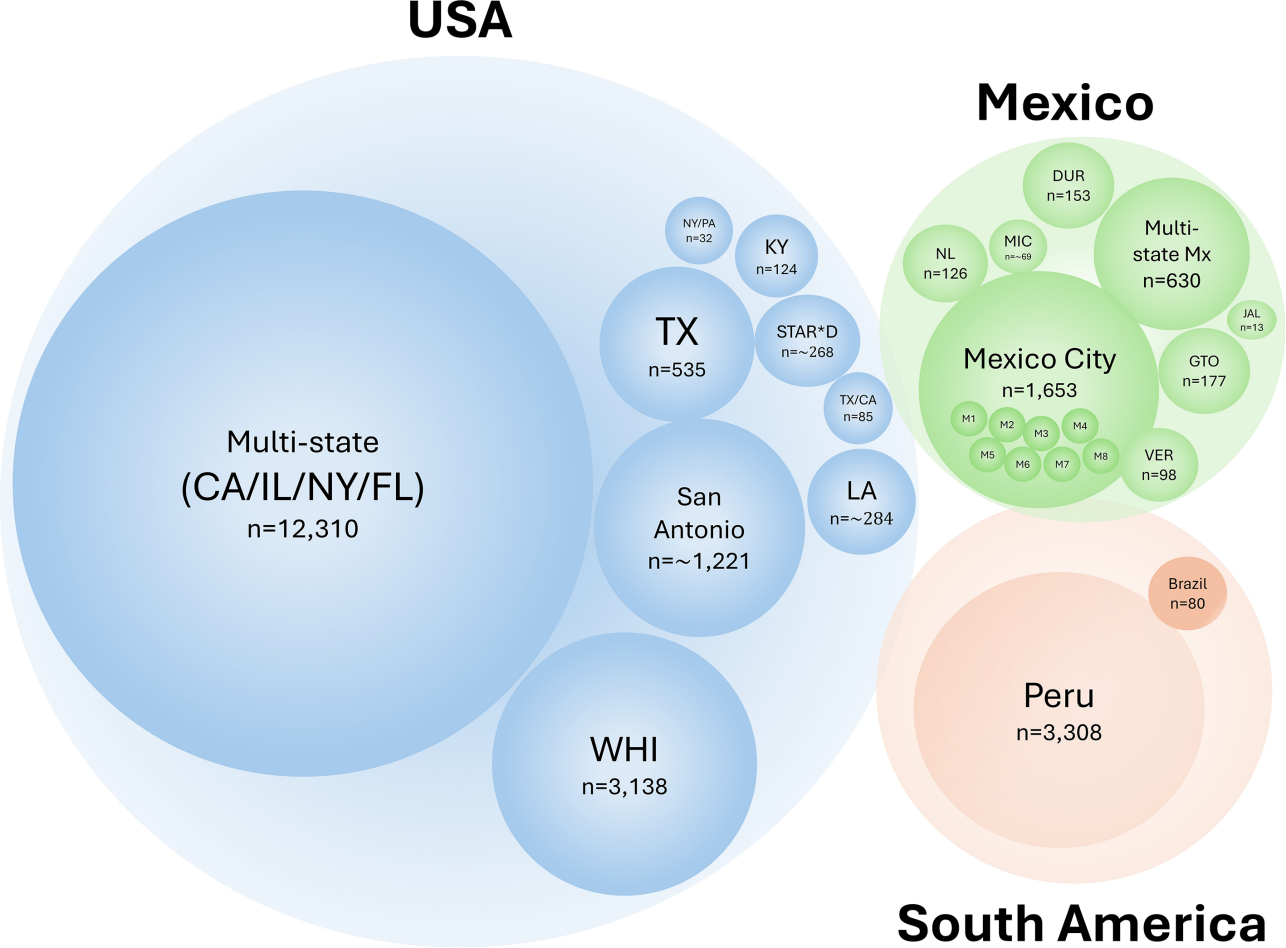
Geographic distribution of shared SNPs among 26 Latino cohorts with depression. Circle size denotes the proportion of the total sample contributed by each cohort; individual Mexico City cohorts are labeled separately and their sample sizes (n) are provided in the caption. The figure summarizes the spatial distribution of all cohorts included in the cross-population analysis of shared SNPs associated with depression. Sample sizes correspond to the maximum reported n for each cohort when multiple publications derived from the same underlying population. Study references and cohort details are provided in **Table 2**. WWHI, Participants in the Women's Health Initiative (WHI) observational study and clinical trials. DUR, Durango. GTO, Guanajuato. JAL, Jalisco. MIC, Michoacan. Multi-State Mx, Diverse Mexican states from the Study of Nutritional and Psychosocial Markers of Frailty. NL, Monterrey. VER, Veracruz. Mexico City cohorts are labeled M1–M8; values indicate sample size (n): M1 = 105, M2 = 33, M3 = 226, M4 = 246, M5 = 197, M6 = 175, M7 = 590, M8 = 81.

Overall, 26 analytically distinct population cohorts were identified across the selected studies (**Figure 2**). These cohorts were used for subsequent analyses of SNP overlap and population-specific reporting.

### 3.3 Genetic variants reported in Latin American populations with depression

As described in the Methods section, we identified shared genetic variants across the 26 analytically distinct population cohorts included in this review (**Figure 2**; **Table 2**). To ensure accurate harmonization across studies, data integration, SNP matching, and cohort-based filtering were performed using R version 4.5.2 and RStudio Desktop. SNP identifiers were standardized using rsIDs, and overlap across datasets was evaluated using scripted, reproducible workflows. When the same SNP was reported in multiple publications derived from the same population cohort, only the original report (i.e., the first publication describing the SNP in that cohort) or the report providing the most detailed genetic information (e.g., allele frequencies or effect estimates) was retained for synthesis. Subsequent publications based on the same cohort were excluded to avoid duplication.

Across all included studies, excluding the genome-wide dataset from Shen et al. (68), a total of 305 distinct SNPs were reported (**Supplementary Table S2**). Among these, eight SNPs were reported in more than one population cohort or publication within the reviewed literature, indicating recurrence across studies included in the systematic review (**Table 3**).

**Table 3:** SNP Characteristics, allele frequencies, molecular effects, and cohort overlap among genotyped Latino participants with depression.

SNP	Gene	Alt allele		Protein	SNP molecular effect	Reference	Cohort	n
		*Total*	*Latino*					
*rs25531*	SLC6A4	C=0.02641, G = 0.00000	C=0.000, G = 0.000	Serotonin transporter (5-HTT)	Reduces promoter activity, making it functionally more similar to the short (S) allele of 5-HTTLPR, leading to lower expression of the serotonin transporter. Can influence serotonin reuptake in the synapse, affecting serotonergic signaling. Individuals carrying the Lg or S alleles tend to have reduced transporter levels, which has been linked to altered stress response, susceptibility to depression, and variable response to SSRIs	(36)	Michoacan, MX	72
						(38)	Dallas TX; Long Beach and Los Angeles, CA, USA	85
						(66)	Brazil	2,392
						(67)	Mexico City, MX	197
						(33)	STAR*D*	196
*rs1549870*	PDE1A	A=0.10630	A=0.0708	Phosphodiesterase 1A (PDE1A)	Reduced PDE1A expression, leading to increased intracellular cyclic nucleotides (cAMP in case of PDE1A and PDE11A; cGMP in case of PDE11A and PDEA9) levels. Increased cyclic nucleotide signaling enhances CREB-mediated transcription of neurotrophic factors such as BDNF, promoting neuronal survival, synaptic plasticity, and neurogenesis in mood-related brain regions. These molecular effects may counteract neuronal deficits associated with depression and contribute to improved antidepressant response observed in carriers of the Alt alleles.	(32)	STAR*D* and Los Angeles, CA, USA*	239** + 268
*rs729861*	PDE9A	G=0.50778	G=0.327	Phosphodiesterase 9A (PDE9A)				
*rs1880916*	PDE11A	A=0.212417	A=0.0708	Phosphodiesterase 11A (PDE11A)				
						(25)	Los Angeles, CA, USA*	284
*rs4680*	COMT	A=0.483863	A=0.41378	Catechol-O-methyltransferase (COM)	Reduces COMT enzyme activity, leading to slower dopamine degradation in the prefrontal cortex. This may increase dopamine availability, influencing stress response and emotional regulation, and has been associated with altered susceptibility to depression, particularly under conditions of chronic stress.	(36)	Michoacan, MX	72
						(37)	STAR*D*	238
						(67)	Mexico City, MX	197
						(58)	Kentucky, USA	124
*rs429358*	APOE	C=0.033403	C=0.0330	Apolipoprotein E (Apo ϵ4)	Alters the protein’s structure and lipid-binding properties, affecting cholesterol transport and metabolism. ϵ4 carriers have a higher risk for Alzheimer’s disease, cardiovascular disease, and differences in neuronal repair. ϵ4 is associated with reduced hippocampal volume and impaired neurogenesis, both implicated in depression vulnerability. APOE ϵ4 may promote higher neuroinflammation and altered stress hormone (cortisol) regulation, which are known contributors to depressive symptoms. Some studies suggest depression in older adults may be an early manifestation of neurodegenerative processes in which APOE ϵ4 plays a role.	(42)	Mexico City, MX	226
						(48)	Brownsville and McAllen TX, USA	1,320
*rs4570625*	TPH2	T=0.236687	T=0.34093	Tryptophan hydroxylase 2 (TPH2)	Reduces promoter activity with lower TPH2 expression and decreased serotonin synthesis. Higher risk for depression, stress reactivity and altered response to SSRIs.	(56)	Veracruz, MX	98
						(50)	Mexico City, MX	96
*rs6265*	BDNF	T=0.183420	T=0.16138	Brain-derived neurotrophic factor (BDNF)	Reduces activity-dependent secretion of BDNF. This leads to impaired synaptic plasticity, neurogenesis, and neuronal survival, particularly in the hippocampus and prefrontal cortex. Carriers of the Alt allele have been associated with increased susceptibility to depression, altered stress response, and potentially reduced response to antidepressant treatments.	(41)	Adolescents from Mexico City, MX	213
						(40)	Los Angeles, CA, USA*	278
*rs510769*	OPRM1	T=0.253462	T=0.3290	μ-opioid receptor (MOR)	Reduced MOR expression (a G protein–coupled receptor), leading to weaker receptor signaling in response to endogenous opioids (like endorphins, enkephalins). This reduction can impair reward processing, stress buffering, and pain modulation, as the μ-opioid system plays a key role in hedonic tone and stress resilience. Individuals with diminished MOR signaling may experience lower mood, reduced ability to feel pleasure (anhedonia), and heightened stress sensitivity. These molecular effects may increase vulnerability to depression and influence responses to treatments targeting the opioid system.	(34)	STAR*D*	163
*rs9322445*		G=0.15416	G=0.113					
*rs6923231*		A=0.07182	A=0.0468					
*rs3778155*		T=0.15612	T=0.119			(68)	Lima, PE	3,308
*rs10485057*		G=0.09822	G=0.079					
*rs645189*		G=0.89054	G=0.913					

STAR*D, Sequenced Treatment Alternatives to Relieve Depression. Included Latinos from Dallas TX, Los Angeles and San Diego, CA, USA. * indicates overlap of cohorts across articles. **Latino subsample size (n=239) was inferred from the total sample reported in the original study; this value was not explicitly confirmed in the published article and should be interpreted with caution. Alt allele: alternate (minor) allele; frequencies shown correspond to the Alt allele. The frequency of the Alt allele for Latinos corresponds to BioSample: SAMN10492700. The Los Angeles Mexican-American cohort may partially overlap with the gnomAD MXL reference population; therefore, allele-frequency comparisons should be interpreted with caution, as full independence cannot be assumed. 5-HTTLPR: Serotonin-Transporter-Linked Polymorphic Region. SSRIs, Selective Serotonin Reuptake Inhibitors. n, number of depressed Latino individuals whose samples underwent genotyping.

In a separate comparison, the 306 SNPs identified in the systematic review were evaluated against genome-wide association summary statistics from Shen et al. (68). This analysis identified 51 SNPs that were also present in the genome-wide dataset (**Supplementary Table S3**). Among these overlapping variants, six SNPs showed nominal statistical significance (P < 0.05) in the genome-wide analysis. These six variants were further evaluated and are reported separately from SNPs that recur within the reviewed cohorts. In total, 14 unique SNPs were identified after systematically excluding duplicate cohort-level evidence, such that each population cohort contributed only once per SNP. These variants represent all SNPs observed across more than one population cohort and do not reflect recurrence driven by multiple publications from the same underlying dataset. Of these, eleven SNPs were reported in studies derived from the STAR*D Latino subpopulation, indicating substantial overlap across analyses using this cohort. The Mexican-American cohort recruited in Los Angeles shared four SNPs with other cohorts. Three SNPs overlapped between the STAR*D and Los Angeles cohorts, while six SNPs were shared between the STAR*D and the Peruvian cohort, suggesting convergence of reported genetic variants across geographically and demographically distinct Latino populations (see **Table 3**).

Our approach focused on SNPs observed in Latin populations with depressive symptoms, prioritizing those replicated across multiple cohorts, regardless of their statistical significance in individual studies. We did not formally distinguish between candidate gene studies and GWAS, as our primary goal was to identify variants showing consistency across cohorts. This strategy was chosen due to the broadly described heterogeneity among existing studies and allows us to strengthen the likelihood of identifying true associations while providing population-specific insights. Among the included studies, Wong et al., 2017 (47); Dunn et al., 2016 and 2018 (53, 54); and Shen et al., 2020 (68) represent GWAS, whereas Yu et al., 2017 (49) corresponds to an exome-wide association study (EWAS). Of these, only Shen et al., 2020 (68) conducted a full GWAS, identifying 1,047,613 SNPs that reached the standard genome-wide significance threshold (P < 5×10^-8^). None of these genome-wide significant SNPs were observed in the other cohorts included in our review.

One recurrent variant, rs25531, was reported across five independent cohorts: the STAR*D Latino cohort from Los Angeles and San Diego, California (33); Mexican-American samples from Texas and California (38), Mexican cohorts from Michoacan (36) and Mexico City (67), and a Brazilian cohort (66). This distribution indicates that rs25531 has been consistently reported across multiple Latin American populations, including both native-country and diaspora cohorts.

Similarly, the SNP rs4680 was identified in four independent cohorts, including two U.S.-based samples (the STAR*D Latino cohort and a Kentucky cohort) (37, 58) and two Mexican cohorts from Michoacan and Mexico City (36, 67). The repeated identification of rs4680 across these cohorts suggests recurrence of this variant in studies of depression among Latino populations.

#### 3.3.1 Variants in monoaminergic pathway genes

Among the SNPs reported across the included studies, a substantial proportion were located in genes involved in monoaminergic neurotransmission. These variants were identified in genes related to serotonin transport and synthesis, and dopamine metabolism, and were reported across multiple independent Latino cohorts. Results for individual monoaminergic genes are detailed in the following subsections.

##### 3.3.1.1 *SLC6A4* (rs25531)

Across the included studies, genetic variation in the serotonin transporter gene (SLC6A4) was evaluated using three analytical frameworks: (1) the biallelic 5-HTTLPR model (L/S) , (2) the genotypic triallelic model incorporating rs25531 (La, Lg, S), and (3) the functional triallelic model grouping alleles as high-expression (L′) or low-expression (S′) (**Table 4A**). Five studies evaluated SLC6A4 variation in Latino populations, encompassing cohorts from the United States, Mexico, and Brazil (**Table 4B**).

**Table 4A:** Functional meaning of 5-HTTLPR/rs25531 alleles.

Allele / Genotype	Molecular definition	Functional expression	rs25531 base
La (Long + A)	L allele with A at rs25531	High-expression allele; efficient SERT transcription (L’= L functional)	A (reference)
Lg (Long + G)	L allele with G at rs25531	Low-expression allele (functionally similar to S; S’ = S functional); altered transcriptional factor binding	G (variant)
L (classic)	Long insertion at 5-HTTLPR (unsubtyped)	Higher expression unless rs25531 = G (then becomes Lg)	—
S (classic)	Short deletion at 5-HTTLPR	Low expression	—
Functional groups (triallelic model)	L’ = La; S’ = S + Lg	Reflects functional transporter expression. S’ = S functional. L’= L functional.	—

HTTLPR, serotonin transporter-linked promoter region. SERT, serotonin transporter.

**Table 4B:** Summary of 5-HTTLPR and rs25531 findings across Latino and admixed populations with depression.

Study	Population	Genotype / allele distribution in latinos (triallelic)	Functional classification used	Main findings
Gudayol-Ferré et al. (36)	72 adults with MDD, Michoacán, Mexico	*La carriers (L’) = 52.8%, non-carriers (S’ = S +Lg) = 47.2%.	Classic and triallelic model: La carriers (L’), and non-carriers (S’ = S or Lg).	rs25531 not associated with early fluoxetine remission. Clinical/psychological factors (comorbid anxiety, cognition) better predicted outcomes. Low Lg frequency.
Pierucci-Lagha et al. (38)	85 Mexican-Americans under IFN-α therapy (plus Caucasians & African Americans)	L’L’ = 24.7%, L’S’ = 41.2%, S’S’ = 34.1%.	Triallelic functional model (La = high, Lg+S = low).	In Latinos with prior depression, SS had strongest increase in depressive symptoms. Clear gene × prior-depression interaction.
Rocha et al. (66)	80 depressed participants, Brazil (Pelotas Birth Cohort)	LL = 23.8%, LS = 40%, SS = 36.2%.	Classic and functional triallelic model for analysis of risk and G×E interactions.	Childhood maltreatment × S allele → higher depression risk, especially in SS. rs25531 inclusion approached significance but not decisive. African ancestry enriched low-expression alleles.
Sanabrais-Jiménez et al. (67)	197 adolescents with MDD, Mexico City	*LL = 13.2%, LS = 44.2%, SS = 42.6%. Allele Frequency: S’ = 64.9%, L’ = 35.1%.	Triallelic functional grouping (La, Lg, S).	Adolescents showed high prevalence of SS. rs25531 not directly associated with depression severity or suicidality.
Mrazek et al. (33)	STAR*D cohort: 196 Mexican-Americans (plus Caucasians & African Americans)	Latinos: La = 93.9%, Lg = 6.1%. African Americans: higher Lg (25.4%).	Focused on rs25531 SNP.	No association between rs25531 genotype and citalopram remission. Clear population-specific allele distributions (Lg rare in Latinos, frequent in African Americans).

Cauc, Caucasians. STAR*D, Sequenced Treatment Alternatives to Relieve Depression. Included Latinos from Dallas TX, Los Angeles and San Diego, CA, USA. IFN-α, Interferon-alpha. GxE, Gene-Environment interaction. HTTLPR, Serotonin Transporter-Linked Promoter Region. MDD, Major Depressive Disorder. *Mexican-American from: Dallas, Texas; San Diego and Los Angeles California form the STAR*D cohort. **Latinos from: Dallas, Texas; Long Beach and Los Angeles California. ^+^, 72 patients were recruited and genotyped while only 64 completed the trial. * Percentages were calculated from raw counts for cross-study comparison.

The included studies differed in design, clinical context, and outcome definitions, including antidepressant response, treatment-emergent depressive symptoms, and clinically defined depression.

In the context of antidepressant response, Mrazek et al. (22) analyzed 196 Mexican-American participants from the STAR*D cohort who were treated with citalopram. No association was observed between the rs25531 genotype and treatment remission in Latinos, Caucasians, or African Americans. Allele distribution differed by ancestry: the A allele (La) was highly prevalent in Latinos (93.9%) and Caucasians (92.9%), but substantially less frequent in African Americans (74.6%). Conversely, the G allele (Lg) was more frequent among African Americans (25.4%) than among Latinos (6.1%) or Caucasians (7.1%).Similarly, Gudayol-Ferré (36) reported 72 adults with major depressive disorder from Michoacan, Mexico. Genotype frequencies for 5-HTTLPR were 28 S/S, 31 S/L, and 13 L/L. Among S/L and L/L individuals, the major carried at least one La allele; however, the number of rs25531 carriers was not explicitly reported. No association was detected between rs25531 and early response to fluoxetine treatment. The study did not specify the total number of rs25531 carriers, thereby limiting interpretation of the role of rs25531 in depression in this cohort.

In a non-psychiatric clinical context, Pierucci-Lagha et al. (38) assessed depressive symptoms during interferon-alpha (IFN-α) therapy in Mexican American patients with chronic hepatitis C. In Latinos (n = 85), the S’ allele frequency was 54.7%, higher than in African Americans (46.7%) and similar to Caucasians (50.8%). While overall genotype distribution did not differ significantly across ethnic groups, pairwise comparisons indicated a higher S’ allele frequency in Latinos than in African Americans, but not more than in Caucasians.

Depressive symptoms increased during IFN-α treatment across all ethnic groups; however, symptom trajectories differed by 5-HTTLPR genotype and ethnicity. Among Latinos with a history of depression, S’ homozygotes showed the greatest symptom increase between weeks 12 and 20 of treatment, heterozygotes showed a modest decrease, and L’ homozygotes demonstrated a marked reduction in symptom severity over the same period. In contrast, African Americans showed no significant genotype x depression history x time effects. Among Caucasians with prior depression, S’ homozygotes exhibited a decrease, heterozygotes no change, and L’ homozygotes a modest increase in depressive symptoms during the same period.

In contrast, Sanabrais-Jiménez et al. (67) analyzed 197 Mexican adolescents with MDD from Mexico City. Functional reclassification revealed a high proportion of low-expression genotypes (42.6% S’S’, 44.1% L’S’, 13.2% L’L’). No direct association was found between the rs25531 genotype and depression severity or suicidal behavior.

Evidence from Brazil further underscores the relevance of environmental context. Rocha et al. (66) studied 80 Brazilian participants from the Pelotas Birth Cohort with a history of depression. Individuals carrying a greater number of short alleles showed increased depression risk following childhood maltreatment, with homozygotes showing the strongest effect. Inclusion of rs25531 in a triallelic model approached statistical significance but did not fully account for the gene x environment interaction. The number of depressed individuals carrying the rs25531 G allele was not reported.

##### 3.3.1.2 *TPH2* (rs4570625)

Two studies conducted in Latino populations evaluated the association between the *TPH2* rs4570625 polymorphism and depressive phenotypes (50, 56). These studies examined distinct clinical contexts, including depressive symptom variation in non-clinical populations and depression occurring in medically treated cohorts.

Hernandez-Mixteco et al. (56) examined a cohort of 98 adults from Veracruz, Mexico (east-central region), without a history of neurological or psychiatric disorders, assessing variation in depressive symptom scores. Genotype frequencies for rs4570625 were 21.43% GG, 59.18% GT, and 19.39% TT. Carrying the TT genotype exhibited lower scores on the Beck Depression Inventory than carriers of the G allele.

In a medically treated cohort, Rojas-Osornio et al. (50) analyzed rs4570625 in Mexican patients living with HIV who were receiving antiretroviral therapy. All participants were treated with tenofovir disoproxil fumarate and emtricitabine in combination with either efavirenz or atazanavir/ritonavir (ATV/r). The prevalence of depression was 90.4% among individuals receiving efavirenz and 87.5% among those receiving ATV/r. The rs4570625 TT genotype was initially associated with two-fold increased risk of severe depression; however, allele distribution was similar between controls and depressed patients (GG: 35.5% vs. 28.5–35.8%; GT: 50.8% vs. 48.7–59.5%; TT: 13.5% vs. 11.9–15.3%).

In the same study, the rs1386493 GG genotype conferred a five-fold increased risk of depression; this variant was not assessed in the cohort examined by Hernandez-Mixteco et al. (56).

##### 3.3.1.3 *COMT* (rs4680)

Among the 45 included in this review, four examined *COMT* rs4680 (Val108/158Met) polymorphism in Latino populations. The included studies evaluated rs4680 across distinct outcome domains, including antidepressant treatment response, clinically diagnosed MDD, and depressive symptom-related phenotypes.

Gudayol-Ferré (36) investigated the role of rs4680 in predicting remission in a cohort of 72 adults with MDD from Michoacan, Mexico, treated with fluoxetine for 12 weeks. Genotype data were available for 56 participants (AA = 9, AG = 26, GG = 21). Of these, 43 individuals (76.7%) reached remission, whereas 13 (23.3%) did not. Non-remitting patients represented a higher number of comorbid anxiety disorders and a trend toward a greater number of previous depressive episodes. Logistic regression analysis identified the AG and GG genotypes as significant predictors of remission, while clinical and neuropsychological variables did not independently contribute to the model.

Ji et al. (37) analyzed the association between rs4680 and antidepressant treatment outcomes in the STAR*D cohort, which included 1,232 Caucasians, 287 African Americans, and 238 Latinos with MDD. No significant association between rs4680 and non-remission was observed in Caucasians (p = 0.449; OR = 1.06; 95% CI = 0.91–1.24), with minor allele frequencies (MAF) of 0.48 in non-remitters and 0.47 in remitters. Similarly, no significant association was detected in African Americans (p = 0.354; OR = 0.84; 95% CI = 0.58–1.21) with MAFs of 0.32 in non-remitters and 0.28 in remitters. In contrast, the Latino subgroup showed a trend toward significance (p = 0.061; OR = 1.48; 95% CI = 0.98–2.24), with differences in allele frequencies between non-remitters (MAF = 0.344) compared to remitters (MAF = 0.429).

Sanabrais-Jiménez et al. (67) examined rs4680 in a cohort of 197 adolescents with MDD from Mexico City. Genotype distribution was relatively balanced, with 58 individuals (29%) carrying Val/Val, 80 (41%) Val/Met, and 50 (25%) Met/Met genotypes. Allele frequencies were approximately 50% for Val and 46% for Met. When stratified by history of suicide attempts, the Met allele was more frequent among adolescents with a history of suicide attempt (60%) compared with non-attempers (42%).

Key et al. (58) examined the relationship between ethnic discrimination, rs4680, and depressive symptoms in U.S. Latinos. The study was conducted in a cohort of 124 adults from Kentucky, USA, a population in which ethnic discrimination is frequently experienced and is linked to cardiovascular disease (CVD) risk factors such as depression. The authors aimed to investigate whether the rs4680 genotype was associated with depressive symptoms severity and whether it moderated the impact of ethnic discrimination. Genotype distribution included 48 Val/Val individuals and 76 combined Met-allele carriers (Val/Met and Met/Met combined). The rs4680 polymorphism was significantly associated with depressive symptom severity (p = 0.016), with Met-allele carriers exhibiting higher depressive symptom scores compared to Val/Val individuals (p = 0.041). Both ethnic discrimination (p = 0.041) and rs4680 genotype (p = 0.049) independently contributed to depressive symptom severity, whereas no significant interaction between discrimination and genotype rs4680 was observed.

Across the four studies, rs4680 was examined in relation to different depression-related outcomes. Two studies evaluated antidepressant treatment response, one study assessed clinically diagnosed MDD and suicidal behavior, and one study analyzed depressive symptom severity in relation to environmental exposure.

#### 3.3.2 Phosphodiesterase gene variants

Among the 14 shared variants analyzed across cohorts, three intronic phosphodiesterase (PDE) SNPs were investigated first in the Los Angeles Mexican-American cohort (25) and these results were then compared against the Latino subset of STAR*D study (32). In both cohorts, participants with clinically MDD were included, and outcomes related to both diagnosis and treatment response were examined.

With respect to antidepressant treatment response, Wong et al. (25) reported that the major genotype of rs1549870 was associated with significantly higher remission rates following fluoxetine, with an odds ratio of 8.8 (95% CI: 1.71–45.24). In this cohort, the A allele frequency was 2.2% among remitters compared with 14% among non-remitters. In contrast, Cabanero et al. (32) observed no significant association between rs1549870 remission following citalopram treatment in Latino participants from STAR*D, in which the A allele frequency was 6.45%.

In analyses examining both MDD diagnosis and treatment response, Wong et al. (25) reported that rs1880916 was associated with both MDD diagnosis and antidepressant remission and identified a PDE11A haplotype (GAACC) that differed significantly between patients and controls (p < 0.0001). Using this cohort, Luo et al. (29) performed an expanded analysis of the chromosome 2q31–q32 region encompassing *PDE1A* and *PDE11A*, examining 22 SNPs. Several *PDE11A* haplotypes showed significantly different frequencies between cases and controls. In addition, individuals carrying the rs1880916 (*PDE11A*) AG/AA genotype in combination with the rs1549870 (*PDE1A*) GG genotype demonstrated higher remission rates following fluoxetine or desipramine treatment.

Regarding MDD diagnosis, the intronic SNP rs729861 was significantly associated in the Mexican-American cohort reported by Wong et al. (25) (p = 0.0006, Bonferroni-corrected), with the A allele occurring more frequently in affected individuals. This association was not reported in the Latino subset of STAR*D by Cabanero et al. (32).

#### 3.3.3 *APOE* variants (rs429358, rs7412)

Our systematic review identified two studies examining the association between *APOE* variants and depression in Latino populations. These studies were conducted in older adult populations and evaluated depression in the context of aging and comorbid conditions. García-Peña et al. (42) evaluated older adults from a Mexico City cohort and reported that depression was associated with multiple factors, including sex, education, number of chronic diseases, anxiety, serious illness (personal or of a close relative), functional limitations, and hypertension. However, no statistically significant associations were observed between depression and *APOE* SNPs rs429358 or rs7412 in this population.

In contrast, Xu et al. (48) reported significant associations between the *APOE*-ϵ4 allele and both anxiety and depression in a Latino population over 60 years old in Texas, USA, using data derived from cohorts that included individuals with neurocognitive conditions. In this study, ϵ4 carriage was associated with increased odds of depression (OR = 1.48, 95% CI 1.13–1.95, p < 0.004). In this study, a higher prevalence of comorbid conditions, including anxiety and Alzheimer’s disease, was observed.

#### 3.3.4 *BDNF* variant (rs6265)

The Brain-derived neurotrophic factor (BDNF) rs6265 (Val66Met) polymorphism has been examined in Latino populations with heterogeneous findings. The included studies evaluated rs6265 in relation to clinically diagnosed MDD and, in some cases, in the context of environmental exposures.

Cruz-Fuentes et al. (41) analyzed 213 adolescents from Mexico City and found no direct association between rs6265 and MDD. Genotype distributions in depressed versus control adolescents were Met/Met 1.9% vs. 2%, Met/Val 21.6% vs. 21.5%, and Val/Val 76.5% vs. 76.4% (p = 0.99). Psychosocial adversities, including abuse, neglect, family dysfunction, parental maladjustment, parental death, and life-threatening illness, were the strongest predictors of depression. Stratified analyses showed that in females, carriers of the Met allele exhibited lower odds of depression in the context of cumulative childhood adversity (OR = 0.2; 95% CI = 0.09–0.7; p < 0.02).

Ribeiro et al. (40) evaluated 278 Latino adults from Los Angeles and reported a significant association between rs6265 and MDD. The G allele (Val) was associated with increased odds of depression, with GG homozygotes showing a 1.7-fold higher risk (95% CI 1.17–2.47; p = 0.005). Among depressed individuals, genotype frequencies were GG 79.1%, AG 20.1%, and AA 0.7%, compared with controls (GG 69.1%, AG 29.1%, AA 1.9%).

#### 3.3.5 *OPRM1* variants

Two studies have investigated the distribution and MDD relevance of μ-opioid receptor (*OPRM1*) polymorphisms in Latino populations (1): Latino participants from the STAR*D cohort residing in Dallas, San Diego, and Los Angeles (34), and (2) individuals with depression from Peru (68). In both studies, the *OPRM1* variants were reported in Supplementary Materials as part of broader candidate-gene or genome-wide analyses, and both applied ancestry-informed analytical approaches. These studies examined OPRM1 variants in relation to both depression-related phenotypes and antidepressant treatment response in Latino populations.

Shen et al. (68) conducted genome-wide association and polygenic risk score analyses in a cohort of 3,308 adult females from a low–socioeconomic status community in Lima, Peru. Participants reported high exposure to adverse life events during childhood and adulthood. Psychiatric phenotypes assessed included depressive symptom severity, post-traumatic stress disorder (PTSD), and suicidal ideation/self-harm. Six *OPRM1* variants showed nominal single-variant associations with depression (*P* < 0.05): rs510769, rs9322445, rs6923231, rs3778155, rs10485057, and rs645189. In parallel, polygenic risk scores derived primarily from European-ancestry cohorts demonstrated significant associations with depression in the Peruvian sample.

Garriock et al. (34) examined 53 *OPRM1* variants in 163 Latino participants with MDD from the STAR*D cohort, evaluating associations with citalopram treatment outcomes measured using QIDS-SR. Treatment response was defined as a ≥50% reduction in symptom severity, with sustained response assessed across follow-up visits. Garriock et al. (34) further reported that, after Bonferroni correction for multiple testing, the strongest association observed was between the *OPRM1* variant rs540825 and specific antidepressant response in Latino participants.

The six *OPRM1* variants nominally associated with depression in the Shen et al. (68) analysis were also evaluated in the STAR*D Latino sample. Among these overlapping variants, rs3778155 showed nominal associations in both studies, whereas the remaining variants reached nominal significance only in the Shen et al. dataset.

### 3.4 Risk of bias assessment

#### 3.4.1 Interventional studies

##### 3.4.1.1 Randomized controlled trials

**Figure 3:**
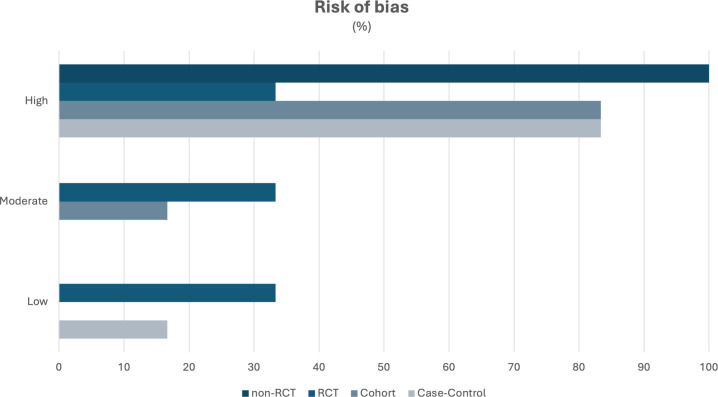
Summary of risk-of-bias assessments. Study quality was evaluated using three design-specific instruments: RoB2 for randomized controlled trials (RCT), ROBINS-I V2 for non-randomized controlled studies (non-RCT), and the Newcastle–Ottawa Scale (NOS) for observational designs (Cohort and Case-control). One study reporting both randomized and non-randomized results was assessed with both RoB2 and ROBINS-I V2 (32). For graphical comparability, final judgments were harmonized into three categories: low risk/high quality, moderate risk/some concerns, and high risk/serious or critical risk/low quality; and expressed as percentages within each study design.

The quality of nine randomized controlled trials (RCTs) was assessed using the RoB 2 tool, including the randomized component of Cabanero et al. (32). One-third of the trials were rated as low risk of bias, one-third as having some concerns, and one-third as high risk of bias (**Figure 3**).

Studies classified as high risk of bias primarily exhibited limitations in the domain of selection of the reported results. Trials rated as having some concerns most frequently presented issues related to deviations from intended interventions, particularly regarding the effect of assignment to intervention. Studies rated as low risk of bias demonstrated adequate performance across the domains of randomization, missing outcome data, and outcome measurement.

Based on overall RoB 2 assessments, Wong et al. (26), Dong et al. (27), and Wu et al. (30) were classified as low risk of bias. Licinio et al. (24), Licinio et al. (28), and Wong et al. (31) were rated as high risk of bias. The remaining trials were classified as having some concerns (25, 29, 32).

##### 3.4.1.2 Non-randomized controlled trials

Seven non-randomized interventional studies were evaluated using the ROBINS-I V2 tool, including the non-randomized component of Cabanero et al. (32). All studies were classified as low quality due to serious risk of bias in at least one ROBINS-I domain (32, 33, 35–38).

The most frequently affected domain was bias due to confounding, followed by bias in the selection of participants into the study or analysis (**Figure 3**).

#### 3.4.2 Observational studies

##### 3.4.2.1 Case-control studies

Among the case–control studies, Newcastle–Ottawa Scale (NOS) scores ranged from 1 to 7 points. Two studies, Camarena et al. and Ribeiro et al. (39, 40), achieved high quality ratings (7 points), although Ribeiro et al. (40) was classified as moderate quality due to limitations in the comparability domain. The remaining studies (n = 11) scored between 1 and 2 points and were classified as low quality.

Low-quality studies commonly showed limitations in the selection domain, including lack of representativeness of cases and unclear definition or selection of controls. All low-quality studies scored 0 points in the comparability domain. Exposure or outcome ascertainment generally scored between 1 and 2 points.

##### 3.4.2.2 Cohort studies

For cohort studies, NOS scores ranged from 1 to 6 points. Three studies, Dunn et al. (54), Rocha et al. (66), and Shen et al. (68), were classified as moderate quality, with scores of 4, 6, and 5 points, respectively. The remaining studies (n = 15) scored between 1 and 3 points and were classified as low quality.

Low-quality cohort studies frequently demonstrated limited adjustment for confounding and insufficient information on outcome assessment or follow-up duration.

## 4 Discussion

Given the heterogeneity of study designs, findings related to gene variants were interpreted within two distinct frameworks: (i) genetic susceptibility to depression and (ii) pharmacogenetic effects on antidepressant response. These domains reflect different biological mechanisms and are therefore discussed separately where applicable.

### 4.1 Monoaminergic pathway variants in Latino populations with depression

Monoaminergic neurotransmitters play central roles in mood regulation. Serotonin is involved in mood, appetite, and sleep; norepinephrine contributes to arousal, attention, and stress responses; and dopamine is implicated in motivation, reward processing, and hedonic experience. Alterations in the synthesis, release, or reuptake of these neurotransmitters have long been implicated in depressive disorders, forming the basis of the classical monoamine hypothesis of depression (69).

Across the included studies, variants affecting serotonin transport, synthesis, and dopamine availability were repeatedly identified in both Latin American and U.S.-based Latino cohorts. Accordingly, the following subsections summarize evidence for key monoaminergic pathway genes. However, the strength of these associations is limited by the predominance of low-quality studies in which those have been reported.

#### 4.1.1 *SLC6A4* (rs25531)

The serotonin (5-hydroxytryptamine, 5-HT) system is frequently investigated in genetic studies of depression due to its established role in mood regulation and its relevance as a pharmacological target. Accordingly, several studies included in this review examined genetic variation within serotonergic pathway genes, particularly the serotonin transporter gene SLC6A4. Dysregulation of 5-HT neurotransmission is implicated in various neuropsychiatric disorders, including MDD (70, 71).

Serotonin signaling is regulated at multiple levels, including synthesis, release, uptake, receptor signaling, and metabolic degradation via monoamine oxidase (MAO). The serotonin transporter (SERT), encoded by the SLC6A4 gene, plays a central role in clearing extracellular serotonin and is directly targeted by SSRIs, the most widely prescribed antidepressants. Expression of SERT is influenced by serotonin-transporter-linked polymorphic region (5-HTTLPR), a 44-base pair insertion/deletion in the promoter region of *SLC6A4*. The long (L) allele is associated with higher transcriptional activity, whereas the short (S) allele confers reduced expression and decreased serotonin transporter availability (72). Reduced 5-HTT expression has been linked to increased susceptibility to depression and altered stress responsivity (73). The rs25531 SNP further refines the functional interpretation of SLC6A4 variation. This adenine-to-guanine substitution within the L allele alters transcription factor binding, including AP2, subdividing the L allele into a high-expression (La) and low-expression (Lg) form. In this triallelic framework, the Lg allele behaves functionally similarly to the S allele, and both are classified as low-expression S’ alleles (72). As a result, rs25531 is essential for accurate functional classification of serotonin transporter expression and for interpreting associations involving 5-HTTLPR.

Although rs25531 (SLC6A4) was assessed in five independent cohorts, differences in genetic models, outcome definitions, and population characteristics precluded formal meta-analysis. Findings are therefore summarized narratively to accurately reflect cohort-specific associations.

##### 4.1.1.1 Genetic susceptibility

Across the studies included in this review, rs25531 did not show a strong or consistent direct association with depression risk in Latino populations. Notably, the available evidence for this variant is largely derived from studies of low methodological quality. Only the study from Rocha et al. (66), received a score of moderate quality.

Accordingly, evidence for genotype-related effects was most apparent when salient environmental or pharmacological stressors were present, including IFN-α exposure among individuals with a prior history of MDD (38) and childhood maltreatment among short-allele homozygotes in cohorts where depressive symptoms were assessed using non-diagnostic indices (66). Collectively, these findings reinforce a gene–environment interaction model in which serotonin transporter variation influences depression-related outcomes primarily through contextual modulation rather than as an independent genetic risk factor in Latinos.

Nevertheless, a coherent pattern emerged when allele distributions and functional classifications were examined in the context of ancestry. Across Latino cohorts, a high prevalence of functionally low-expression alleles (S′) was observed (38, 66, 67) as summarized in **Table 4B**, although this pattern was not driven by enrichment of the rs25531 Lg allele. Instead, studies that explicitly genotyped rs25531 indicated that the low-expression Lg variant was relatively rare in Latino populations, including Mexican cohorts, with the majority of L alleles corresponding to the high-expression La subtype (33, 35).

A direct ancestry comparison available in Mrazek et al. (33) further demonstrated a substantially higher frequency of the Lg allele among African American participants (25.4%) compared with Latinos (6.1%), indicating a clear ancestry-related difference in rs25531 allele distribution. These findings are consistent with prior literature showing that African ancestry is associated with higher frequencies of functionally low-expression alleles, particularly the Lg variant, whereas European and Native American ancestry is characterized by low Lg frequencies despite variable S allele prevalence (74–76). Given that Latino populations are predominantly admixed with European and Native American ancestry and generally exhibit lower African admixture, these results suggest that reduced serotonin transporter expression associated with rs25531 in Latino populations is predominantly mediated by the S allele rather than by enrichment of the Lg variant.

This interpretation is further supported by evidence from a non-clinical Colombian cohort, which reported no association between serotonin-related polymorphisms and depressive symptoms in the absence of environmental stressors (77). Although this study was not included in the systematic search and is cited for contextual comparison only, it underscores the importance of ancestry composition, clinical status, and environmental context when interpreting the role of serotonin transporter variation in mood-related outcomes.

##### 4.1.1.2 Pharmacogenetic effects on antidepressant response

Studies evaluating antidepressant response in Latino populations indicate that clinical variables, rather than rs25531 genotype alone, played a central role in treatment outcomes. In Mexican patients treated with fluoxetine, remission was more strongly associated with factors such as comorbid anxiety and cognitive performance than with rs25531 genotype (36).

Similarly, in the STAR*D cohort, no significant association was observed between rs25531 and citalopram remission across Latino participants (33), despite clear differences in allele distribution by ancestry. These findings suggest that rs25531 does not act as a robust independent predictor of antidepressant response in Latino populations.

Pharmacogenetic studies further suggest that carriers of the S-allele may exhibit poorer SSRI response, likely because the pharmacologic target is already under-expressed (78). Lifelong reduction in SERT expression may influence neurodevelopmental trajectories and stress-response circuitry, potentially enhancing emotional reactivity and susceptibility to depressive episodes (73).

Overall, available evidence indicates that the contribution of this *SLC6A4* SNP to pharmacological response is limited and context-dependent and is likely overshadowed by clinical and demographic factors rather than acting as a primary pharmacogenetic determinant.

#### 4.1.2 *TPH*2 (rs4570625)

Tryptophan hydroxylase-2 (TPH2) is the neuron-specific, rate-limiting enzyme in central serotonin biosynthesis and is predominantly expressed in the raphe nuclei (79). By catalyzing the conversion of tryptophan to 5-hydroxytryptophan, TPH2 directly regulates central serotonergic neurotransmission, making it a biologically plausible candidate gene for mood disorders. Accordingly, genetic variation within *TPH2*, particularly the promoter polymorphisms rs4570625 (G-703T), has been investigated in relation to depression susceptibility.

Although the functional consequences of rs4570625 on *TPH2* transcription are not well established, this variant is located within a promoter region overlapping predicted transcription binding sites. Hernandez-Mixteco et al. (56) noted that the G allele overlaps heat shock factor motifs, whereas the T allele overlaps predicted Cdx transcription factor binding sites, suggesting a potential regulatory role for rs4570625 in modulating *TPH2* expression. However, direct functional validation of these effects remains limited, and the available evidence for this variant is largely derived from studies with low methodological quality.

##### 4.1.2.1 Genetic susceptibility

Across the Latino cohorts reviewed here, findings underscore the context-dependent nature of associations involving *TPH2* variation. Evidence suggests that rs4570625 alone does not fully explain depression risk, particularly in clinical settings characterized by strong biological and psychosocial stressors. In populations affected by chronic conditions such as HIV infection, elevated depression risk appears to be driven primarily by disease-related (neuroinflammation, immune system effects, exacerbation by antiretroviral treatment) and psychosocial mechanisms rather than by single genetic variants. Rojas-Osornio et al. (50) discussed the contribution of clinical and environmental factors, including employment status, immune markers such as CD4 count, and broader social or health-related stressors, as important determinants of depression risk in this population. Within this framework, the authors proposed that *TPH2* genetic variants may act as modulators of individual susceptibility in a context-dependent manner, rather than as primary determinants of depressive outcomes.

Although findings are not fully consistent across populations, accumulating evidence supports a role for *TPH2* polymorphisms in the neurobiological basis of depression through their influence on serotonin synthesis and regulation. Meta-analyses have reported associations between rs4570625 and MDD across diverse populations, including individuals of European, Asian, African American, Native American, and Middle Eastern ancestry (80, 81). Nevertheless, the direction and magnitude of these associations appear to vary across populations and environmental contexts.

Ancestral background may partially account for variability in reported associations. Hernandez- Mixteco et al. (56) interpreted their findings as broadly consistent with studies in Asian and Caucasian populations, where the TT genotype has been associated with reduced stress reactivity and lower depression risk, while the GG genotype has been linked to greater vulnerability. Conversely, other studies have associated the T allele with increased anxiety-related traits and neural alterations, highlighting population-specific and context-dependent effects. Although ancestry proportions were not reported in the original studies, population-level genetic surveys indicate that individuals from the Veracruz region typically exhibit higher European ancestry and lower Native American and African contributions (82), whereas Mexico City populations show substantially (as the sample studied by Rojas-Osornio et al. (50) show substantially higher Native American ancestry (55–65%) and lower European admixture (31–41.8%), with similarly low African ancestry (1.8–3%) (83, 84). Because rs4570625 allele frequencies and their functional effects vary across ancestral backgrounds, differences in admixture may partially account for the divergent associations observed between TPH2 variation and depressive phenotypes across Latino populations.

##### 4.1.2.2 Pharmacogenetic effects on antidepressant response

Currently, no studies included in this review directly evaluated TPH2 rs4570625 as a pharmacogenetic predictor of antidepressant response in Latino populations. Therefore, the discussion of TPH2 in this review is focused entirely on genetic susceptibility and context-dependent risk modulation rather than treatment effects.

#### 4.1.3 *COMT* (rs4680)

Catechol-O-methyltransferase (COMT) is a key enzyme in catecholamine metabolism and is highly expressed in the prefrontal cortex and other brain regions, where it regulates the degradation of dopamine, norepinephrine, and epinephrine (85). Through its role in modulating synaptic dopamine availability, COMT influences cognitive function, emotional regulation, and stress responsivity processes that are centrally implicated in MDD and related affective phenotypes (9). The common functional polymorphism rs4680 (G>A), also known as Val158Met, alters enzymatic activity: the Met allele confers reduced COMT activity and relatively higher synaptic dopamine levels, whereas the Val allele is associated with higher enzymatic activity and more rapid catecholamine degradation (86). These functional differences influence prefrontal dopamine signaling and may shape individual differences in emotional reactivity, stress sensitivity, and vulnerability to mood disorders.

Across Latino populations, available evidence suggests that rs4680 does not act as a deterministic risk factor for depression but may instead function as a susceptibility modifier whose effects depend on clinical context, environmental exposures, and phenotype definition. Overall, rs4680 appears to modulate depression-related traits and treatment response in Latinos; however, these associations are derived exclusively from studies with low methodology quality and should therefore be interpreted with caution.

##### 4.1.3.1 Genetic susceptibility

The Met allele has frequently been associated with heightened emotional reactivity and stress sensitivity, particularly in environments characterized by psychosocial or biological stressors (87). Such functional trade-offs may be particularly relevant in populations exposed to cumulative social and environmental stressors. In this context, Key et al. (58) reported that both perceived discrimination and rs4680 genotype were independently associated with depressive symptoms severity in Latino adults, with no evidence of a genotype x discrimination interaction. Although the number of individuals meeting criteria for clinical depression was low, these findings suggest that COMT-related dopaminergic variation may contribute to affective symptom burden in this population regardless of psychosocial exposure. Given the established links between depressive symptoms, chronic stress, and cardiovascular disease, this pattern supports a potential pathway through which genetic and social vulnerability may converge to influence long-term cardiometabolic risk in Latino populations.

Beyond social stressors in adulthood, developmental context may represent another critical dimension shaping COMT-related vulnerability. Findings from Sanabrais-Jiménez et al. (67) highlight the relevance of rs4680 in adolescents with MDD within Latino populations. Adolescence is a period marked by heightened vulnerability to affective dysregulation, during which dopaminergic signaling plays a central role in emotional control and stress responsivity. Genetic variation in *COMT*, a key regulator of cortical dopamine availability, may therefore exert a stronger influence on depression-related outcomes during this developmental stage. In the Mexican adolescent cohort studied by Sanabrais-Jiménez et al. (67), *COMT* variation was linked to clinically severe manifestations of MDD, supporting the notion that rs4680 may act as a susceptibility factor rather than a general risk allele. These findings underscore the importance of considering developmental stage and population context when interpreting dopaminergic genetic associations with depression.

In Latino populations, the Met allele (G) of rs4680 shows a consistent trend as a susceptibility factor for depressive traits, including higher symptom severity and elevated suicide risk, particularly in adolescents and adults exposed to psychosocial stressors.

##### 4.1.3.2 Pharmacogenetic effects on antidepressant response

In the context of antidepressant treatment, *COMT* variation may also contribute to inter-individual differences in pharmacological response. Evidence from Latino cohorts with MDD supports a potential pharmacogenetic role for rs4680, whereby genotypes associated with higher enzymatic activity may confer a greater likelihood of symptomatic improvement during SSRI treatment. Specifically, Gudayol-Ferré et al. (36) proposed that the AA genotype of rs4680, linked to faster catecholamine degradation, is associated with a higher likelihood of symptomatic remission under fluoxetine treatment, while the presence of G alleles may reduce treatment response, highlighting a potential pharmacogenetic influence on antidepressant efficacy. When these findings are considered alongside results from the multi-ethnic STAR*D cohort, also including participants with MDD, in which no significant associations were observed in Caucasian or African American participants, but a suggestive trend was present in Latinos (37), the overall pattern is consistent with a population-specific modulation of antidepressant response rather than a universal pharmacogenetic effect.

Pharmacogenetic studies indicate that Met-allele carriers may experience differential SSRI treatment outcomes, with some evidence suggesting lower remission rates compared to Val carriers. Variability across studies is influenced by age, clinical phenotype, environmental exposures, and ancestry composition.

##### 4.1.3.3 Integrated interpretation of monoaminergic gene variants

Across these findings, genetic susceptibility effects and pharmacogenetic associations are considered separately, highlighting how monoaminergic variants influence depressive traits and treatment outcomes in context-dependent way. The findings from Latino cohorts highlight a nuanced role for monoaminergic gene variants in depression, with both serotonin and dopamine pathways contributing in context-dependent ways. Functional S’ alleles of the serotonin transporter (*SLC6A4*/rs25531) appear to modulate depressive symptoms and treatment outcomes under specific environmental or pharmacological stressors, such as prior depression, interferon−α therapy, or childhood maltreatment. Similarly, the Met allele of COMT rs4680 is associated with higher depressive symptom severity, increased risk of suicidal behavior, and lower remission rates in Mexican and U.S. Latino populations, whereas Val alleles show relatively protective effects. These genotype–phenotype relationships are influenced by ancestry, as allele distributions reflect varying proportions of European, Native American, and African genetic backgrounds, which determine low− versus high−expression allele frequencies.

Beyond transporter and catecholamine metabolism genes, polymorphisms in *TPH2*, the rate-limiting enzyme in serotonin synthesis, have also been implicated in depression. Meta-analyses indicate that several *TPH2* variants, including rs4570625, are associated with mood disorders and related psychiatric conditions, though with small effect sizes (81, 88). In Latino populations, preliminary evidence suggests that the rs4570625 TT genotype may confer either protective or risk effects depending on clinical and environmental context (50, 56), supporting a modulatory rather than causal role of serotonin synthesis genes in depression vulnerability.

This interpretation is consistent with evidence from Colombian cohorts, where COMT variation did not show direct associations with depressive symptoms in healthy individuals, further supporting the notion that monoaminergic variants alone are insufficient to predict depression in Latin populations. These observations are also consistent with recent work questioning the classical serotonin hypothesis of depression (89, 90). While plasma tryptophan, the precursor for serotonin, is reduced in unmedicated depressed patients, tryptophan depletion in healthy individuals does not consistently lower mood, indicating that decreased serotonin availability is neither necessary nor sufficient to cause clinical depression. However, in individuals recovered from depression, tryptophan depletion can trigger clinically significant mood lowering, suggesting that reductions in serotonin interact with pre-existing neurobiological vulnerabilities to influence relapse risk (89).

### 4.2 Phosphodiesterase gene variants

Beyond monoaminergic neurotransmission, cyclic nucleotide signaling pathways regulated by phosphodiesterases have been implicated in depression and antidepressant response, potentially linking monoaminergic, glutamatergic, and inflammatory mechanisms relevant to treatment resistance (14, 15). In this context, genetic studies in Latino populations have examined variants within *PDE1A*, *PDE11A*, and *PDE9A*, highlighting this pathway as biologically plausible while also revealing substantial heterogeneity in genetic associations across cohorts and treatment paradigms. Notably, some of the available evidence derives from RCTs with moderate methodological quality; however, these findings remain limited and should be interpreted cautiously pending replication in higher-quality studies.

#### 4.2.1 Genetic susceptibility

Evidence from the Mexican-American cohort reported by Wong et al. (25), and subsequently extended by the same research group in Luo et al. (29), suggested that intronic variation in *PDE1A* and *PDE11A* may influence MDD susceptibility. Luo et al. (29), identified a *PDE11A* haplotype (GAACC) strongly associated with MDD, while combined-genotype analysis indicated that multi-locus configurations spanning *PDE1A* and *PDE11A* were more informative than individual variants. *PDE9A* rs729861 was initially associated with MDD risk in Wong’s Mexican-American cohort, though this association was not replicated in Latino STAR*D participants (32). Overall, these findings suggest that PDE variants may modulate depression risk, but effects are context- and ancestry-dependent.

#### 4.2.2 Pharmacogenetic effects on antidepressant response

In terms of treatment outcomes, Wong et al. (25) observed marked differences in allele distribution for rs1549870 (*PDE1A*) between fluoxetine remitters and non-remitters. Multi-locus configurations spanning *PDE1A* and *PDE11A* were associated were associated with higher remission rates following fluoxetine or desipramine treatment (29). Together, these findings raised the possibility that coordinated regulation of cAMP and cGMP signaling may modulate antidepressant response, consistent with known roles of cyclic nucleotide pathways in limbic brain regions (91, 92). However, subsequent studies failed to replicate these associations across other populations and treatment settings. In the Latino subset of STAR*D, Cabanero et al. (32) reported no association between rs1549870 and citalopram response, despite an intermediate A allele frequency (6.45%) relative to the Mexican-American cohort.

This frequency notably exceeds population-based estimates for individuals of Mexican ancestry in Los Angeles reported by gnomAD (~0.04%), suggesting possible enrichment of the minor allele in clinical samples or unaccounted ancestry heterogeneity. Similarly, analyses in predominantly European-ancestry samples treated with duloxetine found no association for rs1549870 or rs1880916 (93), and additional STAR*D analyses reported largely null results, with only nominal association in the African American subgroup (94). Collectively, these findings suggest that the original associations may reflect treatment-specific, ancestry-dependent, or haplotype-driven effects rather than broadly generalizable predictors of antidepressant response.

In contrast to the inconsistent findings for *PDE1A* or *PDE11A*, *PDE9A* has emerged as a potentially more robust candidate across study designs. Nevertheless, more recent genome-wide studies have identified *PDE9A* variants as significantly associated with antidepressant remission in depressed older adults with European ancestry (95), suggesting that *PDE9A* may play a broader role in treatment response that is not captured by earlier candidate-gene approaches in Latinos.

#### 4.2.3 Integrated interpretation of *PDE* variants

Overall, the available evidence indicates that PDE variants are present in Latino populations with MDD but exert effects that are highly contingent on ancestry composition, antidepressant class, and analytic resolution. The haplotype-based findings reported by Luo et al. (29) support the notion that multi-marker configurations within PDE genes may be more informative than individual SNPs, while allele-frequency discrepancies relative to gnomAD underscore the importance of careful population characterization. Future studies integrating multi-ancestry genetic designs with functional annotation and pharmacological stratification will be essential to determine whether variants such as rs1549870 (*PDE1A*), rs1880916 (*PDE11A*), and rs729861 (*PDE9A*) contribute meaningfully to antidepressant response or instead represent population-specific markers without direct functional impact.

From a biological perspective, sustained interest in PDE genes is supported by experimental evidence demonstrating that phosphodiesterase inhibition modulates key signaling pathways implicated in mood regulation, including the cAMP–PKA–CREB and cGMP–PKG cascades, while also attenuating neuroinflammation, reducing oxidative stress, and promoting synaptic plasticity (96). Despite this compelling mechanistic framework, no clinical trials have directly examined inhibitors targeting *PDE1A*, *PDE9A*, or *PDE11A* in depressive disorders, leaving the functional and therapeutic relevance of the associated intronic variants unresolved.

### 4.3 *APOE* variants (rs429358, rs7412)

*APOE* encodes apolipoprotein E, a lipid transport protein highly expressed in the liver and central nervous system, where it plays a key role in cholesterol metabolism, neuronal repair, and synaptic plasticity. In the brain, *APOE* isoforms modulate amyloid-β clearance, neuroinflammation, and neuronal resilience, thereby influencing vulnerability to neurodegenerative and mood disorders, including Alzheimer Disease (AD) and MDD (97). The two coding SNPs rs429358 (C>T) and rs7412 (T>C) define the three major *APOE* isoforms (ϵ2, ϵ3, and ϵ4). The C allele at rs429358 results in an arginine substitution at codon 112, altering protein conformation and lipid-binding properties, giving rise to the ϵ4 isoform, which is associated with less efficient lipid transport and impaired neuronal repair (81). These structural changes may affect synaptic function and stress response pathways, providing a molecular basis for the observed association between *APOE*-ϵ4 and increased susceptibility to depression and cognitive decline (81, 98). In contrast, the T allele at rs7412 introduces a cysteine at codon 158, influencing receptor-binding and lipid metabolism, and contributes to the ϵ2 isoform. However, the available evidence in Latino populations is derived exclusively from studies with low NOS scores and should be interpreted cautiously.

#### 4.3.1 Genetic susceptibility

Within this biological framework, the limited and heterogeneous evidence available in Latino populations suggests that the relationship between *APOE* variants and depression is highly context dependent. Differences in comorbidity burden, age distribution, and clinical characteristics across cohorts likely play a central role in shaping the detectability of genetic effects. In particular, the presence of co-occurring anxiety or neurodegenerative conditions may amplify the influence of *APOE*-ϵ4 on depressive phenotypes, whereas its impact may be less apparent in populations with lower comorbidity burden or reduced statistical power.

Genetic ancestry further complicates interpretation. Older adults from central and southern Mexico, such as those represented in Mexico City cohorts, typically exhibit a high proportion of Native American ancestry (55-65%), moderate European admixture (31-41.8%), and minimal African ancestry (1.8-3%). Populations originating from northern Mexican states such as Tamaulipas, Nuevo León, Coahuila, and Chihuahua exhibit higher European ancestry (55%) and slightly greater African input (5%) (82, 84, 99). Latinos residing in Texas, many of whom trace their ancestry to these northern regions, likely share this more European-shifted genetic profile.

Consistent with this gradient, González et al. (100) reported heterogeneity in APOE ϵ-allele frequencies among U.S. Latinos, with ϵ4 ranging from 17.5% in Dominicans to approximately 11% in Mexicans, Central Americans, and South Americans. The higher ϵ4 frequency among Dominicans may reflect their greater proportion of African ancestry, consistent with reports that African American populations exhibit ϵ4 frequencies of 25–30%, substantially higher than those observed in Europeans (32–37%) and Mexican-derived populations (48). These ancestry-dependent differences in rs429358 and rs7412 distributions, together with the small number and modest sample sizes of available studies, likely contribute to the inconsistent associations between *APOE* isoforms and depression observed to date.

Overall, current evidence indicates that *APOE* variation may influence depression risk in Latino populations primarily as a modifier of vulnerability in specific clinical and demographic contexts rather than as a universal genetic risk factor. Future studies incorporating larger sample sizes, detailed comorbidity profiling, and ancestry-informed analyses will be essential to clarify whether *APOE*-ϵ4 contributes directly to depression risk or acts indirectly through its established roles in neurodegeneration and stress-related neurobiology.

#### 4.3.2 Pharmacogenetic effects on antidepressant response

No studies included in this review directly evaluated APOE variants (rs429358 or rs7412) as predictors of antidepressant treatment response in Latino populations. Therefore, the role of APOE in this context remains unclear.

### 4.4 *BDNF* variant *(rs6265)*

BDNF is a key regulator of neuronal survival, synaptic plasticity, and neurogenesis in brain regions critically involved in mood regulation, including the hippocampus and prefrontal cortex (101, 102). The functional rs6265 polymorphism (Val66Met) alters activity-dependent BDNF secretion and has been extensively investigated in relation to depression, although findings across populations remain heterogeneous.

As reviewed by Yang et al. (12), multiple studies conducted predominantly in Caucasian populations have linked the Met (A) allele to increased vulnerability to depressive symptoms, heightened stress sensitivity, and reduced neuroplasticity. However, the same body of literature also includes studies reporting no significant association between rs6265 and depression, suggesting that this variant alone may be insufficient to confer depression risk. In Latino populations, the available evidence is limited and derived from studies with heterogeneous methodological quality, including both high (40) and low (41) NOS scores. This variability further complicates interpretation and suggests that reported associations should be considered with caution pending replication in additional well-characterized cohorts.

#### 4.4.1 Genetic susceptibility

Evidence from Latino populations with MDD reviewed in the present study suggests a pattern that diverges from the canonical Met-risk model described in many Caucasian cohorts. The Met allele appears to be neutral or potentially protective in Mexican adolescents, particularly in females and under conditions of environmental adversity (41); whereas the Val allele may confer increased risk in Los Angeles Latino adults (40).

Although differences in age, environmental exposure, and study design limit direct comparability, the direction observed in this cohort contrasts with that commonly reported in European ancestry samples and further supports population-specific modulation of rs6265 effects. These differences may again reflect ancestry-related patterns, similar to those observed for other molecules: Mexican adolescents from Mexico City are predominantly of Mesoamerican-European admixture (65% Native American, 31% European, and 3% African), whereas Latinos in Los Angeles have a more heterogeneous admixture of Native American (45%), European (49%) and African ancestries (5%) (82–84). Such population-specific genetic backgrounds, along with age and environmental exposures, likely contribute to the divergent associations observed between rs6265 and depression.

Supporting this interpretation, González-Giraldo et al. (77), reported no association between rs6265 and depressive symptoms in a Colombian cohort, further highlighting heterogeneity within Latin American populations themselves.

Overall, the available evidence suggests that BDNF rs6265 should not be regarded as a universal risk variant for MDD. Rather, it may function as a context-dependent modulator of susceptibility or resilience, with effects shaped by ancestry, environmental exposures, sex, and developmental timing. These findings emphasize the need for larger, well-characterized studies in diverse and underrepresented populations, incorporating detailed environmental and ancestry information, to clarify the role of rs6265 in depression risk.

#### 4.4.2 Pharmacogenetic effects on antidepressant response

No studies included in this review directly evaluated rs6265 as a predictor of antidepressant treatment response in Latino populations. Therefore, its pharmacogenetic relevance in this context remains unclear.

### 4.5 *OPRM1* variants

The μ-opioid receptor (MOR), encoded by the *OPRM1* gene, is a G protein-coupled receptor widely expressed in the central nervous system, particularly in brain regions involved in mood regulation, stress response, and reward processing, including the prefrontal cortex, amygdala, and nucleus accumbens. MOR modulates neurotransmission by inhibiting adenylate cyclase activity, reducing cAMP levels, and regulating calcium and potassium channels, ultimately controlling neuronal excitability and synaptic plasticity. Dysregulation of the MOR system has been implicated in the pathophysiology of depression, influencing emotional reactivity, stress sensitivity, and anhedonia (11, 103, 104).

In Latino populations, the available evidence for OPRM1 variants is limited and derives from studies with mixed methodological quality, including a nRCT with low methodological quality (34) and a cohort study with moderate quality (68). This combination of study designs and quality levels constrains the strength of the evidence, and reported associations should therefore be interpreted cautiously pending replication in higher-quality and independent cohorts.

#### 4.5.1 Genetic susceptibility

In this review, six *OPRM1* variants were identified in both in Latino participants with MDD from the STAR*D study and individuals with depressive symptoms from the Peruvian cohort, indicating that these loci are present and polymorphic across Latino populations with markedly different ancestry compositions. Despite differences in admixture proportions (greater European and African ancestry in U.S. Latino cohorts and higher Indigenous American ancestry in the Peruvian sample), the recurrence of these variants suggests a shared involvement of the μ-opioid receptor pathway across Latino populations, rather than population-specific restriction of these loci.

Among the overlapping variants, rs3778155 showed nominal associations in both datasets, whereas the remaining variants reached nominal significance only in the Peruvian cohort. While these findings do not establish replication, they point to rs3778155 as a locus observed across independent Latino samples and distinct phenotypic contexts. Given the modest effect sizes and nominal significance levels, these results should be interpreted cautiously and highlight the need for larger, ancestry-diverse samples to clarify the contribution of *OPRM1* variation in depression-related phenotypes.

Notably, the study by Shen et al. (68) was the only investigation reviewed here to provide comprehensive genome-wide association study (GWAS) data from the South American population. This contribution represents an important step toward addressing the underrepresentation of non-European populations in psychiatric genetics and expanding the availability of genetic resources from low- and middle-income countries. This is particularly relevant in light of current evidence indicating that depression is a highly polygenic trait, with risk distributed across numerous variants of small effect, most of which have been identified in predominantly European-ancestry cohorts (105). In addition to single-variant analyses, Shen et al. (68) demonstrated that polygenic risk scores derived primarily from European-ancestry cohorts were significantly associated with depression-related outcomes in the Peruvian sample, supporting partial sharing of polygenic liability across populations. The Peruvian cohort consisted exclusively of women, a group with higher prevalence rates of depression and trauma-related disorders. The identification of shared *OPRM1* variants in this female-only sample adds relevant context for future studies examining sex-stratified genetic effects, particularly in pathways related to stress and affect regulation.

#### 4.5.2 Pharmacogenetic effects on antidepressant response

Importantly, Garriock et al. (34) reported that, after Bonferrioni correction for multiple testing, the strongest association observed was between the *OPRM1* variant rs540825 and specific antidepressant response in Latino participants. This finding underscores the relevance of the μ-opioid receptor pathway in treatment-related outcomes, suggesting a pharmacogenetic contribution of *OPRM1* variation to antidepressant efficacy.

#### 4.5.3 Integrated interpretation

Collectively, these findings emphasize the importance of ancestry-aware and sex-aware approaches in psychiatric genetics and underscore the value of expanding GWAS efforts in underrepresented populations. While *OPRM1* variants appear to contribute to depression-related phenotypes across Latino populations, their effects are modest, context-dependent, and may extend to both susceptibility and treatment response, warranting further investigation in larger and more diverse cohorts.

### 4.6 Risk of bias assessment

Across all included studies, the majority demonstrated substantial methodological limitations, with low-quality ratings predominating in both case–control and cohort designs. Common weaknesses included poor comparability due to limited adjustment for confounding variables, inadequate reporting of selection procedures and representativeness, and insufficient information regarding follow-up or outcome assessment. Only six studies achieved moderate quality (25, 29, 32, 54, 66, 68), and six studies achieved high quality (26, 27, 30, 39, 40) across observational and interventional designs, underscoring the need for improved methodological rigor in genetic association studies involving Latin American populations.

## 5 Final remarks

The evidence examined indicates that genetic variation across monoaminergic, neurotrophic, lipid-signaling, and intracellular second-messenger pathways contributes to depression susceptibility and treatment response in Latin American populations. These effects are modest and context-dependent.

Variations in diagnostic criteria, sample composition, and analytical approaches further complicate interpretation. The lack of explicit diagnostic differentiation between MDD and PDD further complicates interpretation, as these conditions likely involve partially distinct biological mechanisms and clinical trajectories. This reinforces the need for biologically informed stratification, in which participants are grouped based on relevant genetic variants, ancestry structure, sex-specific neurobiology, and exposure to environmental stressors rather than symptoms *per se*. Age is also critical, as developmental timing shapes vulnerability to adversity; for instance, childhood maltreatment can induce enduring epigenetic alterations in stress-regulatory pathways, increasing depression risk in adulthood. Stratifying cohorts along these biological and developmental dimensions reduces phenotypic heterogeneity, clarifies variable response patterns, and enables more precise assessment of genetic effects. In practice, this involves shifting from broad clinical groupings to biologically grounded subtypes that more accurately reflect underlying mechanisms.

At a molecular level, a subset of variants appears recurrent across independent Latin cohorts. These include *SLC6A4* (5-HTTLPR/rs25531), *BDNF* (rs6265), and *COMT* (rs4680, Val158Met), as well as selected phosphodiesterase loci (*PDE1A*, *PDE11A*, and *PDE9A*). Variants in *TPH2*, particularly rs4570625, have also been examined in Mexican populations, showing ancestry- and context-dependent associations with depressive symptoms and stress sensitivity. These genes converge on serotonergic signaling, dopaminergic tone, and intracellular cAMP/cGMP regulation, mechanisms central to mood and antidepressant response. However, associations are not uniform. For instance, the *COMT* Met allele has been linked to increased depressive symptoms and poorer SSRI outcomes in some Mexican and Mexican-American samples, yet effect sizes vary, and replication outside these groups is inconsistent. *PDE* variants showed significant associations in certain Los Angeles-based cohorts but were not consistently reproduced elsewhere. These findings highlight plausible biological pathways, but clinical translation is premature.

Ancestry plays a key moderating role. Allele frequencies and linkage patterns differ across populations with distinct mixtures of Native American, European, and African ancestry. Failure to model fine-scale ancestry risks attributing population structure effects to biological causation. The transferability of genetic findings across admixed populations is limited, partly due to differences in linkage disequilibrium structure and ancestry composition, as well as the reduced performance of polygenic risk scores derived from predominantly European-ancestry cohorts (106). This concern is amplified by the potential partial overlap between the Los Angeles Mexican-American clinical cohorts and the gnomAD “MXL” reference group. Any allele-frequency comparisons between these sets should therefore be interpreted with caution, as independence cannot be assumed.

Additional complexity arises from gene × environment and gene × sex interactions. Evidence from Toledo-Lozano et al. (45) shows that the *MAO-A* rs1465107 and *MAO-B* rs1799836 alleles interact with adverse childhood experiences to influence MDD severity, with stronger effects in women for rs1465107 and sex-specific patterns for rs1799836. Earlier work by Camarena et al. (39) similarly reported sexually dimorphic associations for *MAO-A* rs1137070 in OCD with comorbid MDD, it is important to note this is a high-quality study in terms of risk of bias. These studies indicate that *MAO-*related risk is likely conditional and shaped by stress exposure and sex-specific neurobiology. Consistent with this conditional effect, García-Peña et al. (42) found that women represented a disproportionately high percentage of participants with depressive symptoms (84.4% women vs. 15.6% men), and that depression was associated with chronic diseases, anxiety, and functional limitations, although the presence of *APOE* SNPs within this subpopulation or the proportion of comorbid conditions was not specified, a limitation common in SNP studies. A similar pattern of context dependence is observed for *APOE* and *OPRM1*. Variants in *APOE* show mixed associations with depressive symptoms, particularly in older adults, suggesting age-related changes in lipid metabolism and neuroinflammation that may vary by ancestry. Likewise, *OPRM1* variants, which affect endogenous opioid signaling, appear to influence stress response and antidepressant effects, but current evidence remains limited and requires replication in diverse cohorts.

Despite variation in outcomes, a shared pattern emerges. Several SNPs appear repeatedly in Latino populations (**Table 3**) with distinct geographic and ancestral backgrounds. This suggests possible common biological pathways or selective pressures. These signals are preliminary but indicate promising targets for further study.

Overall, most studies were conducted by research groups in the United States, with substantial overlap across cohorts (**Table 2**), resulting in only nine truly independent samples. In contrast, studies originating from Mexico contributed 15 unique cohorts. The predominance of Mexican and Mexican-American cohorts likely reflect both the distribution of available studies and the use of country-specific indexing terms in bibliographic databases. Additionally, studies not meeting inclusion criteria were not part of the systematic synthesis but were occasionally cited to support contextual interpretation. The use of a limited number of databases may have restricted the identification of additional relevant studies. Furthermore, the risk-of-bias assessment revealed marked variability in study design, diagnostic approaches, and reporting practices across all studies (**Figure 3**). A critical limitation of this review is that the majority of included studies were of low methodological quality, as reflected by low Newcastle-Ottawa Scale scores and serious risk-of-bias ratings across non-randomized designs. Accordingly, all reported genetic associations should be interpreted as hypothesis-generating rather than confirmatory and are not suitable for clinical application without replication in higher-quality, well-controlled studies.

This synthesis is therefore subject to an additional limitation related to partial overlap of underlying cohorts, particularly the recurrent use of the STAR*D Latino subpopulation and the Los Angeles Mexican-American cohort. Although strict criteria were applied to avoid duplication, retaining only one report per SNP per cohort, this concentration of evidence may still influence the apparent recurrence of certain variants. Accordingly, the 14 SNPs identified as “unique” were defined based on their presence across independent population cohorts after removal of duplicate cohort-level evidence, rather than repeated analyses of the same dataset. Nonetheless, the limited number of distinct cohorts contributing disproportionately to the evidence base warrants cautious interpretation, and observed cross-cohort convergence should be considered preliminary rather than definitive biological validation.

Although none of the included studies explicitly defined Persistent Depressive Disorder (PDD) or assessed symptom persistence over time, some cohorts may have included individuals with chronic depressive symptoms. However, the lack of longitudinal phenotypic characterization precludes differentiation between episodic and persistent forms of depression. This represents an important limitation, particularly given that chronic depressive phenotypes may involve distinct genetic and neurobiological mechanisms. Future studies incorporating longitudinal designs and standardized diagnostic criteria will be essential to determine whether specific genetic variants are associated with persistent forms of depression.

An additional source of heterogeneity arises from the inclusion of studies in which depression was not the primary outcome but rather a secondary measure or comorbidity. These studies were included because they reported relevant genetic data in Latino populations with depressive symptoms; however, their inclusion may reduce the specificity of the review with respect to depression-focused associations. Overall, findings from studies with depression as a primary outcome were broadly comparable to general patterns observed. Nevertheless, this aspect should be taken into account when interpreting the results.

A formal sensitivity analysis restricted to studies with depression as the primary outcome was considered. However, this approach was not pursued, as a substantial proportion of the included studies are derived from a limited number of partially overlapping cohorts, particularly U.S.-based Mexican-American samples and the STAR*D dataset. As a result, restricting the analysis to these studies would not yield an independent subset but rather a re-analysis of highly related samples, limiting the interpretability of such an approach. Nevertheless, a qualitative comparison of studies in which depression was the primary outcome (specifically MDD diagnosis) indicated that the overall pattern of findings remained consistent with the full dataset. Key variants in *SLC6A4*, *COMT*, *BDNF*, *OPRM1*, and phosphodiesterase genes remained implicated, suggesting that the inclusion of studies with depression as a secondary outcome did not drive the primary conclusions of this review. Notably, variants in *APOE* and *TPH2* were not represented within studies in which depression was the primary outcome, but rather in cohorts where depressive symptoms were assessed in the context of aging, neurological conditions, or chronic medical illness. As such, associations involving these variants should be interpreted with caution, as they may reflect context-dependent effects related to comorbidity, treatment exposure, or underlying disease processes rather than primary genetic susceptibility to depression.

These methodological inconsistencies limit comparability across studies, impede a valid meta-analysis, and weaken the overall strength of the evidence regarding causal inference and clinical applicability.

However, movement toward precision psychiatry requires improved methodological coordination. Future research should prioritize:

- Recruitment of diverse and underrepresented Latin American populations. Many existing studies disproportionately represent Mexican or Mexican-American cohorts, while countries with substantial Native American, Afro-Latin American, and mixed-ancestry populations remain minimally studied. Expanding geographic and ancestral representation is essential to prevent biased inferences and ensure clinical applicability across the region.
- Harmonized diagnostic and clinical phenotyping strategies. Variations in diagnostic instruments, symptom severity scales, and the lack of standardized diagnostic definitions, particularly regarding MDD and PDD, introduce substantial heterogeneity. Standardizing diagnostic criteria and clinical characterization will reduce misclassification and allow more reliable comparisons across studies.
- Systematic modeling of ancestry structure and environmental exposures. Depression risk and treatment response are shaped by gene-ancestry and gene-environment interactions. Incorporating ancestry-informative markers and structured assessment of environmental factors, such as childhood adversity, socioeconomic stress, and trauma, can clarify whether associations reflect biological effects or population structure.
- Replication of candidate variants in independent cohorts. Many reported associations remain study-specific and unreplicated. Replication in separate, well-characterized Latin American samples is necessary to determine whether findings are robust, population-specific, or artifacts of sampling or analysis. Given the predominance of studies with low methodological quality and associated risk of bias, the current evidence base is insufficient for clinical translation, and reported findings should be considered preliminary pending validation in higher-quality studies.
- Functional assays to clarify biological impact. Statistical associations alone cannot establish causal mechanisms. Experimental evaluation, such as gene expression analyses, receptor binding studies, and cellular or animal models, is needed to determine how specific variants alter neurotransmission, neuroplasticity, or stress-response pathways.
- Standardized procedures for reporting SNP data and effect direction. Uniform reporting of genotype frequencies, ancestry composition, effect sizes, and directionality of risk or treatment response will improve comparability across studies and enable future meta-analyses. Without standardized reporting, synthesis across populations remains limited. To improve reproducibility and enable future meta-analyses, genetic association studies should adopt standardized reporting practices in line with STREGA recommendations, including the consistent reporting of genotype counts, allele frequencies, effect sizes (OR with 95% CI), and explicit genetic models. This is particularly critical in admixed populations such as those in Latin America, where population stratification may bias associations if not properly addressed (107).

Achieving robust replication will require coordinated, multi-site research efforts across Latin America. Establishing collaborative networks that recruit comparable clinical cohorts across regions would enable consistent phenotyping and genotyping strategies, while shared data repositories and harmonized analytic pipelines would facilitate pooled and meta-analytic evaluation. This need is amplified by the highly polygenic architecture of depression and the persistent underrepresentation of Latin American populations in large-scale genomic studies. Integrating these genetic data with epigenetic (e.g., DNA methylation, histone modification) and transcriptomic profiles will also be critical, as these molecular layers capture dynamic, experience-dependent regulatory changes that static genotypes cannot. Such multi-layered approaches will help distinguish true biological effects from ancestry structure and environmental confounding, identify biologically coherent subgroups, and support the development of precision medicine strategies in diagnosis and treatment, where therapeutic decisions align with individual genetic background, ancestral context, stress history, developmental stage, and current molecular state rather than broad categorical labels of depression.

## Data Availability

Publicly available datasets were analyzed in this study. The dataset used corresponds to supplementary GWAS data published by Shen et al. (68) and is available at: https://static-content.springer.com/esm/art%3A10.1038%2Fs41386-020-0603-5/MediaObjects/41386_2020_603_MOESM4_ESM.txt.

## Supplementary material

Supplementary Table 1 Full search strategies used for study identification. Complete search strings used in PubMed, EBSCO, and Web of Science to identify studies on SNPs and depression in Latin American populations, including keywords and Boolean operators, applied filters and limits (e.g., language, publication type, clinical studies), and exclusion criteria (e.g., reviews, pre-clinical studies, non-human research).

Supplementary Table 2 SNPs identified in Latino individuals with depression across included studies. *Indicates studies reporting the same SNP in the same cohort; these were not considered separate cohorts and are grouped with the original study.

Supplementary Table 3 SNPs identified in the Peruvian cohort that overlap with variants reported in the included studies. *Nominal *P* values < 0.05 as reported in Shen et al. (68).
