# Supplementary material for: Genetic variation associated with depression in Latin American populations: a systematic review of single-nucleotide variants
Source: Front Psychiatry. 2026 Jun 12;17:1811491. doi: 10.3389/fpsyt.2026.1811491 (PMC13307694; doi:10.3389/fpsyt.2026.1811491)
Supplement: Supplementary Table 1 — Full search strategies used for study identification. Complete search strings used in PubMed, EBSCO, and Web of Science to identify studies on SNPs and depression in Latin American populations, including keywords and Boolean operators, applied filters and limits (e.g., language, publication type, clinical studies), and exclusion criteria (e.g., reviews, pre-clinical studies, non-human research). [file Table1.docx]

**Supplementary Table S1. Full search strategies used for study identification.** Complete search strings used in PubMed, EBSCO, and Web of Science to identify studies on SNPs and depression in Latin American populations, including keywords and Boolean operators, applied filters and limits (e.g., language, publication type, clinical studies), and exclusion criteria (e.g., reviews, pre-clinical studies, non-human research).

| **Database** | **Full Search Strategy** | **Filters Applied** | **Date Last Searched** |
| --- | --- | --- | --- |
| **EBSCO** | ((Mexico OR Latin OR Hispanic OR “Central America” OR “South America” OR Mexican) AND (depression OR “depressive disorder” OR “major depressive disorder” OR “persistent depressive disorder”) AND (mutation OR polymorphism OR delete OR insert OR mutant OR “candidate genes” OR “genetic variant” OR “genome-wide association study” OR GWAS)) | Clinical studies only; Language: English or Spanish; Source type: Articles | January 31^st^ 2025 |
| **PubMed** | ((Mexico OR Latin[tiab] OR Hispanic[tiab] OR “Central America”[tiab] OR “South America”[tiab] OR Mexican[tiab]) AND (depression[tiab] OR “depressive disorder”[tiab] OR “major depressive disorder”[tiab] OR “persistent depressive disorder”[tiab]) AND (mutation[tiab] OR polymorphism[tiab] OR delete[tiab] OR insert[tiab] OR mutant[tiab] OR “candidate genes”[tiab] OR “genetic variant”[tiab] OR “genome-wide association study”[tiab] OR GWAS[tiab])) NOT (review[pt] OR “pre-clinical”[tiab])) | Language: English or Spanish; Article type: Articles, Clinical trial  Exclusions: NOT review[pt]; NOT (animal[mh] NOT human[mh]) | January 31^st^ 2025 |
| **Web of Science** | ((Mexico OR Latin OR Hispanic OR “Central America” OR “South America” OR Mexican) AND (depression OR “depressive disorder” OR “major depressive disorder” OR “persistent depressive disorder”) AND (mutation OR polymorphism OR delete OR insert OR mutant OR “candidate genes” OR “genetic variant” OR “genome-wide association study” OR GWAS)) | Language: English or Spanish; Document type: Articles | January 2025 |

**Supplementary Table S2. SNPs identified in Latino individuals with depression across included studies.** * Indicates studies reporting the same SNP in the same cohort; these were not considered separate cohorts and are grouped with the original study.

| **No.** | **Reference** | **SNP** |
| --- | --- | --- |
| 1 | Mrazek et al. 2009, Gudayol-Ferré et al. 2010*, Gudayol-Ferré et al. 2012, Pierucci-Lahga et al. 2010, Rocha et al. 2015 and Sanabrais-Jimenez et al. 2021 | rs25531 |
| 2 | Gudayol-Ferré et al. 2010*, Gudayol-Ferré et al. 2012, Ji et al. 2012, Sanabrais-Jimenez et al. 2021 and Key et al. 2022 | rs4680 |
| 3 | Ribeiro et al. 2007, Licinio et al. 2009*, Wong et al. 2012*, Cruz-Fuentes et al. 2014 | rs6265 |
| 4 | Hernandez-Mixteco et al. 2023 and Rojas-Osornio et al. 2024 | rs4570625 |
| 5 | Wong et al. 2006; Cabanero et al., 2009 and Luo et al. 2009* | rs1549870 |
| 6 | Wong et al. 2006; Cabanero et al., 2009 and Luo et al. 2009* | rs1880916 |
| 7 | García-Peña et al., 2010 and Xu et al. 2023 | rs429358 |
| 8 | Cabanero et al., 2009 and Wong et al. 2006 | rs729861 |
| 9 | Garriock et al. 2010 and Shen et al. 2020 | rs510769 |
| 10 | Garriock et al. 2010 and Shen et al. 2020 | rs9322445 |
| 11 | Garriock et al. 2010 and Shen et al. 2020 | rs6923231 |
| 12 | Garriock et al. 2010 and Shen et al. 2020 | rs3778155 |
| 13 | Garriock et al. 2010 and Shen et al. 2020 | rs10485057 |
| 14 | Garriock et al. 2010 and Shen et al. 2020 | rs645189 |
| 15 | Ringman et al. 2004* and Ringman et al. 2005* | rs63750083 |
| 16 | Wong et al. 2008* and Wong et al. 2012* | rs4603 |
| 17 | Wong et al. 2008* and Wong et al. 2012* | rs1043307 |
| 18 | Wong et al. 2008* and Wong et al. 2012* | rs115668237 |
| 19 | Wong et al. 2008* and Wong et al. 2012* | rs140395831 |
| 20 | Wong et al. 2008* and Wong et al. 2012* | rs142029931 |
| 21 | Wong et al. 2008* and Wong et al. 2012* | rs142151549 |
| 22 | Wong et al. 2008* and Wong et al. 2012* | rs150952348 |
| 23 | Wong et al. 2008* and Wong et al. 2012* | rs200520741 |
| 24 | Wong et al. 2008* and Wong et al. 2012* | rs200897153 |
| 25 | Wong et al. 2008* and Wong et al. 2012* | rs201483250 |
| 26 | Wong et al. 2008* and Wong et al. 2012* | rs201935337 |
| 27 | Wong et al. 2008* and Wong et al. 2012* | rs2228078 |
| 28 | Wong et al. 2008* and Wong et al. 2012* | rs3744550 |
| 29 | Wong et al. 2008* and Wong et al. 2012* | rs3770018 |
| 30 | Wong et al. 2008* and Wong et al. 2012* | rs41310573 |
| 31 | Wong et al. 2008* and Wong et al. 2012* | rs56293203 |
| 32 | Wong et al. 2008* and Wong et al. 2012* | rs56344012 |
| 33 | Wong et al. 2008* and Wong et al. 2012* | rs701865 |
| 34 | Wong et al. 2008* and Wong et al. 2012* | rs748441912 |
| 35 | Wong et al. 2008* and Wong et al. 2012* | rs782472239 |
| 36 | Wong et al. 2008* and Wong et al. 2012* | rs78562453 |
| 37 | Wong et al. 2017* and Yu et al. 2017* | rs112610420 |
| 38 | Wong et al. 2017* and Yu et al. 2017* | rs115054458 |
| 39 | Wong et al. 2017* and Yu et al. 2017* | rs62001028 |
| 40 | Wong et al. 2017* and Yu et al. 2017* | rs143696449 |
| 41 | Cabanero et al., 2009 | rs3770016 |
| 42 | Cabanero et al., 2009 | rs4893975 |
| 43 | Cabanero et al., 2009 | rs6433687 |
| 44 | Camarena et al., 2001 | rs1137070 |
| 45 | Carless et al., 2011 | rs12137417 |
| 46 | Carless et al., 2011 | rs4658966 |
| 47 | De la Cruz-Ahumada et al. 2023 | rs1800796 |
| 48 | De la Cruz-Ahumada et al. 2023 | rs1800896 |
| 49 | Dong et al., 2009 | rs10276036 |
| 50 | Dong et al., 2009 | rs11140793 |
| 51 | Dong et al., 2009 | rs1128503 |
| 52 | Dong et al., 2009 | rs1362621 |
| 53 | Dong et al., 2009 | rs140701 |
| 54 | Dong et al., 2009 | rs1624327 |
| 55 | Dong et al., 2009 | rs17064 |
| 56 | Dong et al., 2009 | rs2013566 |
| 57 | Dong et al., 2009 | rs2032583 |
| 58 | Dong et al., 2009 | rs2066713 |
| 59 | Dong et al., 2009 | rs2214103 |
| 60 | Dong et al., 2009 | rs2235020 |
| 61 | Dong et al., 2009 | rs2289657 |
| 62 | Dong et al., 2009 | rs2289658 |
| 63 | Dong et al., 2009 | rs2550936 |
| 64 | Dong et al., 2009 | rs28914831 |
| 65 | Dong et al., 2009 | rs3730276 |
| 66 | Dong et al., 2009 | rs3813034 |
| 67 | Dong et al., 2009 | rs3842 |
| 68 | Dong et al., 2009 | rs45596934 |
| 69 | Dong et al., 2009 | rs4728697 |
| 70 | Dong et al., 2009 | rs4783899 |
| 71 | Dong et al., 2009 | rs5564 |
| 72 | Dong et al., 2009 | rs56142442 |
| 73 | Dong et al., 2009 | rs56355214 |
| 74 | Dong et al., 2009 | rs58898486 |
| 75 | Dong et al., 2009 | rs7020204 |
| 76 | Dong et al., 2009 | rs7038236 |
| 77 | Dong et al., 2009 | rs7212502 |
| 78 | Dong et al., 2009 | rs7224199 |
| 79 | Dong et al., 2009 | rs8179029 |
| 80 | Dong et al., 2009 | rs9969765 |
| 81 | Dunn et al., 2016 | rs10166852 |
| 82 | Dunn et al., 2016 | rs10227305 |
| 83 | Dunn et al., 2016 | rs10249677 |
| 84 | Dunn et al., 2016 | rs10886733 |
| 85 | Dunn et al., 2016 | rs1129411 |
| 86 | Dunn et al., 2016 | rs11738766 |
| 87 | Dunn et al., 2016 | rs13033587 |
| 88 | Dunn et al., 2016 | rs16823787 |
| 89 | Dunn et al., 2016 | rs17345417 |
| 90 | Dunn et al., 2016 | rs2282123 |
| 91 | Dunn et al., 2016 | rs2532087 |
| 92 | Dunn et al., 2016 | rs2822657 |
| 93 | Dunn et al., 2016 | rs2912513 |
| 94 | Dunn et al., 2016 | rs34359572 |
| 95 | Dunn et al., 2016 | rs35612712 |
| 96 | Dunn et al., 2016 | rs4542757 |
| 97 | Dunn et al., 2016 | rs58707171 |
| 98 | Dunn et al., 2016 | rs609508 |
| 99 | Dunn et al., 2016 | rs61848143 |
| 100 | Dunn et al., 2016 | rs61973969 |
| 101 | Dunn et al., 2016 | rs6579218 |
| 102 | Dunn et al., 2016 | rs6736484 |
| 103 | Dunn et al., 2016 | rs9601962 |
| 104 | Dunn et al., 2018 | rs10228308 |
| 105 | Dunn et al., 2018 | rs10611601 |
| 106 | Dunn et al., 2018 | rs1127233 |
| 107 | Dunn et al., 2018 | rs112849100 |
| 108 | Dunn et al., 2018 | rs116270819 |
| 109 | Dunn et al., 2018 | rs12198471 |
| 110 | Dunn et al., 2018 | rs13417200 |
| 111 | Dunn et al., 2018 | rs139550651 |
| 112 | Dunn et al., 2018 | rs139628768 |
| 113 | Dunn et al., 2018 | rs140262007 |
| 114 | Dunn et al., 2018 | rs144133307 |
| 115 | Dunn et al., 2018 | rs146173796 |
| 116 | Dunn et al., 2018 | rs186703566 |
| 117 | Dunn et al., 2018 | rs187816263 |
| 118 | Dunn et al., 2018 | rs189585011 |
| 119 | Dunn et al., 2018 | rs2004237 |
| 120 | Dunn et al., 2018 | rs34208798 |
| 121 | Dunn et al., 2018 | rs374519284 |
| 122 | Dunn et al., 2018 | rs4985364 |
| 123 | Dunn et al., 2018 | rs56712382 |
| 124 | Dunn et al., 2018 | rs62123284 |
| 125 | Dunn et al., 2018 | rs6603149 |
| 126 | Dunn et al., 2018 | rs7198462 |
| 127 | Dunn et al., 2018 | rs72662446 |
| 128 | Dunn et al., 2018 | rs74495014 |
| 129 | Dunn et al., 2018 | rs77219363 |
| 130 | Dunn et al., 2018 | rs902168 |
| 131 | Galfalvy et al., 2009 | rs1800532 |
| 132 | Galfalvy et al., 2009 | rs4537731 |
| 133 | García-Peña et al., 2010 | rs7412 |
| 134 | Garriock et al. 2010 | rs10485058 |
| 135 | Garriock et al. 2010 | rs1067685 |
| 136 | Garriock et al. 2010 | rs1067693 |
| 137 | Garriock et al. 2010 | rs12203621 |
| 138 | Garriock et al. 2010 | rs12209447 |
| 139 | Garriock et al. 2010 | rs13195018 |
| 140 | Garriock et al. 2010 | rs1323041 |
| 141 | Garriock et al. 2010 | rs1323044 |
| 142 | Garriock et al. 2010 | rs17174794 |
| 143 | Garriock et al. 2010 | rs17209711 |
| 144 | Garriock et al. 2010 | rs17210667 |
| 145 | Garriock et al. 2010 | rs1799971 |
| 146 | Garriock et al. 2010 | rs2010884 |
| 147 | Garriock et al. 2010 | rs2075572 |
| 148 | Garriock et al. 2010 | rs34427887 |
| 149 | Garriock et al. 2010 | rs3778151 |
| 150 | Garriock et al. 2010 | rs3798683 |
| 151 | Garriock et al. 2010 | rs477292 |
| 152 | Garriock et al. 2010 | rs4870266 |
| 153 | Garriock et al. 2010 | rs499796 |
| 154 | Garriock et al. 2010 | rs505340 |
| 155 | Garriock et al. 2010 | rs510587 |
| 156 | Garriock et al. 2010 | rs524731 |
| 157 | Garriock et al. 2010 | rs538174 |
| 158 | Garriock et al. 2010 | rs540825 |
| 159 | Garriock et al. 2010 | rs553202 |
| 160 | Garriock et al. 2010 | rs557748 |
| 161 | Garriock et al. 2010 | rs562859 |
| 162 | Garriock et al. 2010 | rs563649 |
| 163 | Garriock et al. 2010 | rs583664 |
| 164 | Garriock et al. 2010 | rs589046 |
| 165 | Garriock et al. 2010 | rs606148 |
| 166 | Garriock et al. 2010 | rs610231 |
| 167 | Garriock et al. 2010 | rs613341 |
| 168 | Garriock et al. 2010 | rs618207 |
| 169 | Garriock et al. 2010 | rs620496 |
| 170 | Garriock et al. 2010 | rs649840 |
| 171 | Garriock et al. 2010 | rs658156 |
| 172 | Garriock et al. 2010 | rs679987 |
| 173 | Garriock et al. 2010 | rs686633 |
| 174 | Garriock et al. 2010 | rs7772959 |
| 175 | Garriock et al. 2010 | rs9322446 |
| 176 | Hodgson et al. 2017 | rs7932341 |
| 177 | Ji et al. 2012 | rs4633 |
| 178 | Ji et al. 2012 | rs11569716 |
| 179 | Ji et al. 2012 | rs13306278 |
| 180 | Ji et al. 2012 | rs165599 |
| 181 | Ji et al. 2012 | rs165722 |
| 182 | Ji et al. 2012 | rs165774 |
| 183 | Ji et al. 2012 | rs174696 |
| 184 | Ji et al. 2012 | rs2020917 |
| 185 | Ji et al. 2012 | rs3810595 |
| 186 | Ji et al. 2012 | rs45454096 |
| 187 | Ji et al. 2012 | rs4646310 |
| 188 | Ji et al. 2012 | rs4646315 |
| 189 | Ji et al. 2012 | rs4646316 |
| 190 | Ji et al. 2012 | rs4818 |
| 191 | Ji et al. 2012 | rs5031015 |
| 192 | Ji et al. 2012 | rs5993883 |
| 193 | Ji et al. 2012 | rs737866 |
| 194 | Ji et al. 2012 | rs740603 |
| 195 | Ji et al. 2012 | rs769224 |
| 196 | Ji et al. 2012 | rs9332377 |
| 197 | Ji et al. 2012 | rs9332381 |
| 198 | Ji et al. 2012 | rs933271 |
| 199 | Knowles et. al. 2016 | rs1123988 |
| 200 | Knowles et. al. 2016 | rs11245316 |
| 201 | Knowles et. al. 2016 | rs4578341 |
| 202 | Knowles et. al. 2016 | rs4995180 |
| 203 | Knowles et. al. 2016 | rs7095366 |
| 204 | Knowles et. al. 2016 | rs7906808 |
| 205 | Knowles et. al. 2016 | rs7906939 |
| 206 | Knowles et. al. 2016 | rs7913161 |
| 207 | Licinio et al. 2004 | rs1876828 |
| 208 | Licinio et al. 2004 | rs242939 |
| 209 | Licinio et al. 2004 | rs242941 |
| 210 | Licinio et al. 2009 | rs11030101 |
| 211 | Licinio et al. 2009 | rs11030103 |
| 212 | Licinio et al. 2009 | rs12273539 |
| 213 | Licinio et al. 2009 | rs28722151 |
| 214 | Licinio et al. 2009 | rs41282918 |
| 215 | Luo et al. 2009 | rs2037757 |
| 216 | Malacara et al. 2004 | rs2234693 |
| 217 | Malacara et al. 2004 | rs9340799 |
| 218 | Mathias et al. 2016 | rs12455524 |
| 219 | Mathias et al. 2016 | rs1815955 |
| 220 | Mathias et al. 2016 | rs3910708 |
| 221 | Mathias et al. 2016 | rs648105 |
| 222 | Mathias et al. 2016 | rs8094926 |
| 223 | Mathias et al. 2016 | rs976655 |
| 224 | Mathias et al. 2016 | rs9966747 |
| 225 | Moctezuma et al., 2024 | rs27027 |
| 226 | Moctezuma et al., 2024 | rs7970177 |
| 227 | Rabaneda‑Bueno et al. 2021 | rs2476601 |
| 228 |  |  |
| 229 | Rojas-Osornio et al. 2024 | rs1386493 |
| 230 | Rojas-Osornio et al. 2024 | rs7305115 |
| 231 | Sanabrais-Jimenez et al. 2021 | rs6275 |
| 232 | Sandoval-Carrillo et al. 2018 | rs1799724 |
| 233 | Sandoval-Carrillo et al. 2018 | rs1800629 |
| 234 | Sandoval-Carrillo et al. 2018 | rs361525 |
| 235 | Serna-Rodriguez et al. 2024 | rs4900442 |
| 236 | Serna-Rodriguez et al. 2024 | rs754203 |
| 237 | Toledo-Lozano et al. 2023 | rs1465107 |
| 238 | Toledo-Lozano et al. 2023 | rs1799836 |
| 239 | Wong et al. 2006 | rs220818 |
| 240 | Wong et al. 2006 | rs2544934 |
| 241 | Wong et al. 2006 | rs30585 |
| 242 | Wong et al. 2006 | rs370013 |
| 243 | Wong et al. 2006 | rs3775845 |
| 244 | Wong et al. 2006 | rs650058 |
| 245 | Wong et al. 2006 | rs676389 |
| 246 | Wong et al. 2006 | rs717602 |
| 247 | Wong et al. 2006 | rs884162 |
| 248 | Wong et al. 2006 | rs992185 |
| 249 | Wong et al. 2008 | rs1002205 |
| 250 | Wong et al. 2008 | rs10904481 |
| 251 | Wong et al. 2008 | rs1202186 |
| 252 | Wong et al. 2008 | rs160841 |
| 253 | Wong et al. 2008 | rs17244587 |
| 254 | Wong et al. 2008 | rs1922243 |
| 255 | Wong et al. 2008 | rs2057168 |
| 256 | Wong et al. 2008 | rs2057169 |
| 257 | Wong et al. 2008 | rs2118404 |
| 258 | Wong et al. 2008 | rs2231449 |
| 259 | Wong et al. 2008 | rs2242480 |
| 260 | Wong et al. 2008 | rs2281740 |
| 261 | Wong et al. 2008 | rs2296840 |
| 262 | Wong et al. 2008 | rs2325717 |
| 263 | Wong et al. 2008 | rs34095 |
| 264 | Wong et al. 2008 | rs3744483 |
| 265 | Wong et al. 2008 | rs3746651 |
| 266 | Wong et al. 2008 | rs3809758 |
| 267 | Wong et al. 2008 | rs3817629 |
| 268 | Wong et al. 2008 | rs41515744 |
| 269 | Wong et al. 2008 | rs798412 |
| 270 | Wong et al. 2008 | rs798416 |
| 271 | Wong et al. 2008 | rs852977 |
| 272 | Wong et al. 2008 | rs917195 |
| 273 | Wong et al. 2012 | rs1065756 |
| 274 | Wong et al. 2012 | rs1208 |
| 275 | Wong et al. 2012 | rs2229125 |
| 276 | Wong et al. 2012 | rs2234926 |
| 277 | Wong et al. 2012 | rs2302339 |
| 278 | Wong et al. 2012 | rs3744793 |
| 279 | Wong et al. 2012 | rs4318 |
| 280 | Wong et al. 2012 | rs4648072 |
| 281 | Wong et al. 2012 | rs4723010 |
| 282 | Wong et al. 2012 | rs4988496 |
| 283 | Wong et al. 2012 | rs6205 |
| 284 | Wong et al. 2012 | rs6267 |
| 285 | Wong et al. 2012 | rs7690296 |
| 286 | Wong et al. 2014 | rs1321744 |
| 287 | Wong et al. 2014 | rs16867321 |
| 288 | Wong et al. 2014 | rs3729931 |
| 289 | Wong et al. 2014 | rs658382 |
| 290 | Wong et al. 2017 | rs112002983 |
| 291 | Wong et al. 2017 | rs201138159 |
| 292 | Wu et al. 2011 | rs1110400 |
| 293 | Wu et al. 2011 | rs11547464 |
| 294 | Wu et al. 2011 | rs1805005 |
| 295 | Wu et al. 2011 | rs1805006 |
| 296 | Wu et al. 2011 | rs1805007 |
| 297 | Wu et al. 2011 | rs1805008 |
| 298 | Wu et al. 2011 | rs1805009 |
| 299 | Wu et al. 2011 | rs2228479 |
| 300 | Wu et al. 2011 | rs3212364 |
| 301 | Wu et al. 2011 | rs3212366 |
| 302 | Wu et al. 2011 | rs3212367 |
| 303 | Wu et al. 2011 | rs3212368 |
| 304 | Wu et al. 2011 | rs34158934 |
| 305 | Wu et al. 2011 | rs5885479 |
| 306 | Wu et al. 2011 | rs2228478 |

**Supplementary Table S3. SNPs identified in the Peruvian cohort that overlap with variants reported in the included studies.** * Nominal P values < 0.05 as reported in Shen et al. (2020).

| **No.** | **Reference** | **SNP** |
| --- | --- | --- |
| 1 | Key et al. 2022; Sanabrais-Jimenez et al. 2021; Ji et al. 2012; Gudayol-Ferré et al. 2010; Gudayol-Ferré et al. 2012 | rs4680 |
| 2 | Cruz-Fuentes et al. 2014; Licinio et al. 2009; Ribeiro et al. 2007; Wong et al. 2012 | rs6265 |
| 3 | Wong et al. 2006; Wong et al. 2012 | rs701865 |
| 4 | De la Cruz-Ahumada et al. 2023 | rs1800896 |
| 5 | Dunn et al., 2016 | rs13033587 |
| 6 | Dunn et al., 2016 | rs2282123 |
| 7 | Dunn et al., 2016 | rs34359572 |
| 8 | Dunn et al., 2016 | rs35612712 |
| 9 | Dunn et al., 2016 | rs609508 |
| 10 | Dunn et al., 2016 | rs61848143 |
| 11 | Dunn et al., 2018 | rs10228308 |
| 12 | Dunn et al., 2018 | rs34208798 |
| 13 | Rojas-Osornio et al. 2024 | rs1386493 |
| 14 | Rojas-Osornio et al. 2024 | rs7305115 |
| 15 | Garriock et al. 2010 | rs10485057* |
| 16 | Garriock et al. 2010 | rs13195018 |
| 17 | Garriock et al. 2010 | rs17209711 |
| 18 | Garriock et al. 2010 | rs2010884 |
| 19 | Garriock et al. 2010 | rs3778151 |
| 20 | Garriock et al. 2010 | rs3778155* |
| 21 | Garriock et al. 2010 | rs477292 |
| 22 | Garriock et al. 2010 | rs499796 |
| 23 | Garriock et al. 2010 | rs510769* |
| 24 | Garriock et al. 2010 | rs524731 |
| 25 | Garriock et al. 2010 | rs538174 |
| 26 | Garriock et al. 2010 | rs540825 |
| 27 | Garriock et al. 2010 | rs553202 |
| 28 | Garriock et al. 2010 | rs557748 |
| 29 | Garriock et al. 2010 | rs583664 |
| 30 | Garriock et al. 2010 | rs589046 |
| 31 | Garriock et al. 2010 | rs606148 |
| 32 | Garriock et al. 2010 | rs610231 |
| 33 | Garriock et al. 2010 | rs613341 |
| 34 | Garriock et al. 2010 | rs620496 |
| 35 | Garriock et al. 2010 | rs645189* |
| 36 | Garriock et al. 2010 | rs649840 |
| 37 | Garriock et al. 2010 | rs686633 |
| 38 | Garriock et al. 2010 | rs6923231* |
| 39 | Garriock et al. 2010 | rs7772959 |
| 40 | Garriock et al. 2010 | rs9322445* |
| 41 | Ji et al. 2012 | rs4646316 |
| 42 | Dong et al., 2009 | rs2066713 |
| 43 | Dong et al., 2009 | rs2289658 |
| 44 | Dong et al., 2009 | rs9969765 |
| 45 | Licinio et al. 2009 | rs11030101 |
| 46 | Licinio et al. 2009 | rs12273539 |
| 47 | Wong et al. 2006 | rs3775845 |
| 48 | Wong et al. 2006 | rs676389 |
| 49 | Wong et al. 2006 | rs992185 |
| 50 | Wong et al. 2012 | rs6205 |
| 51 | Wong et al. 2014 | rs3729931 |
